# Detection and Exclusion of False-Positive Molecular Formula Assignments via Mass Error Distributions in UHR Mass Spectra of Natural Organic Matter

**DOI:** 10.1021/acs.analchem.4c00489

**Published:** 2024-06-13

**Authors:** Shuxian Gao, Elaine K. Jennings, Limei Han, Boris P. Koch, Peter Herzsprung, Oliver J. Lechtenfeld

**Affiliations:** † Department Environmental Analytical Chemistry, Research Group BioGeoOmics, Helmholtz Centre for Environmental Research—UFZ, Permoserstr. 15, Leipzig D-04318, Germany; ‡ Department of Biosciences, Ecological Chemistry, Helmholtz Centre for Polar and Marine Research—AWI, Am Handelshafen 12, Bremerhaven D-27570, Germany; § University of Applied Sciences, An der Karlstadt 8, Bremerhaven 27568, Germany; ∥ Department Lake Research, Helmholtz Centre for Environmental Research—UFZ, Brückstr. 3a, Magdeburg D-39114, Germany; ⊥ ProVIS–Centre for Chemical Microscopy, Helmholtz Centre for Environmental Research—UFZ, Permoserstr. 15, Leipzig D-04318, Germany

## Abstract

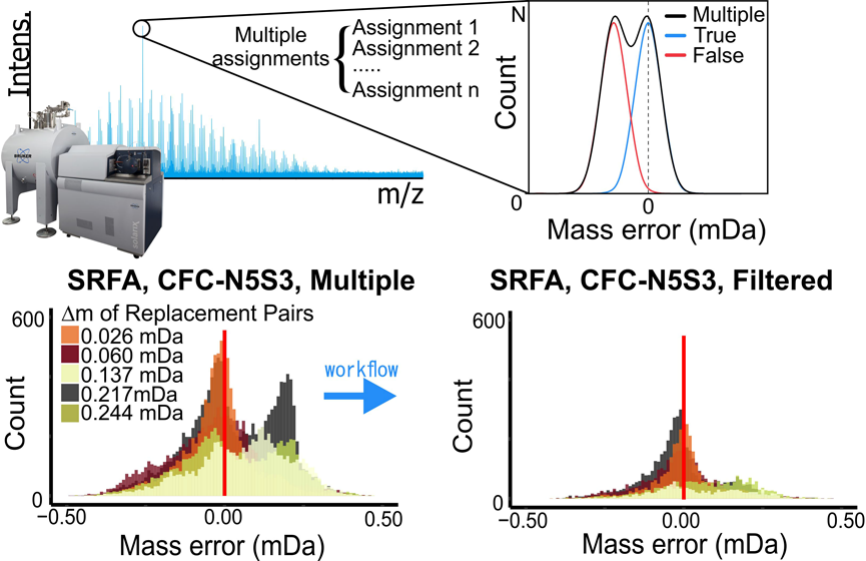

Ultrahigh resolution mass spectrometry (UHRMS) routinely detects and identifies thousands of mass peaks in complex mixtures, such as natural organic matter (NOM) and petroleum. The assignment of several chemically plausible molecular formulas (MFs) for a single accurate mass still poses a major problem for the reliable interpretation of NOM composition in a biogeochemical context. Applying sensible chemical rules for MF validation is often insufficient to eliminate multiple assignments (MultiAs)—especially for mass peaks with low abundance or if ample heteroatoms or isotopes are included - and requires manual inspection or expert judgment. Here, we present a new approach based on mass error distributions for the identification of true and false assignments among MultiAs. To this end, we used the mass error in millidalton (mDa), which was superior to the commonly used relative mass error in ppm. We developed an automatic workflow to group MultiAs based on their shared formula units and Kendrick mass defect values and to evaluate the mass error distribution. In this way, the number of valid assignments of chlorinated disinfection byproducts was increased by 8-fold as compared to only applying ^37^Cl/^35^Cl isotope ratio filters. Likewise, phosphorus-containing MFs can be differentiated against chlorine-containing MFs with high confidence. Further, false assignments of highly aromatic sulfur-containing MFs (“black sulfur”) to sodium adducts in negative ionization mode can be excluded by applying our approach. Overall, MFs for mass peaks that are close to the detection limit or where naturally occurring isotopes are rare (e.g., ^15^N) or absent (e.g., P and F) can now be validated, substantially increasing the reliability of MF assignments and broadening the applicability of UHRMS analysis to even more complex samples and processes.

## Introduction

Ultrahigh resolution mass spectrometry (UHRMS) provides extraordinary resolving power and mass accuracy in the sub parts-per-million (ppm) range. This enables highly accurate mass-to-charge ratio determinations, efficient molecular formula (MF) assignments and identification of compounds without tandem MS experiments.^1−4^ The most powerful UHRMS technique, Fourier transform ion cyclotron resonance mass spectrometry (FT-ICR MS) routinely identifies thousands of MFs from complex mixtures, which enables detailed characterization of crude oil, metabolomics, and natural organic matter (NOM).^5−8^

UHRMS with superior mass resolving power facilitates enhanced separation and identification of closely spaced mass peaks in the mass spectrum, consequently leading to improved mass accuracy and more confident MF assignment.^5^

Even with the high mass accuracy of modern high-field FT-ICR instruments and advanced data processing,^9−11^ multiple MF assignments for the same accurate mass occur within measurement error ranges.^12−15^ Multiple assignments (MultiAs) consist of one common core MF but different “formula residuals”, referred to as *replacement pairs* hereafter. For instance, MFs with a ^12^C_5_^13^C_1_O_4_ residual (e.g., ^12^C_c_^13^C_1_H_h_N_0_O_o_S_0_) could be also assigned with a H_3_N_5_S_2_ residual (^12^C_c–5_^13^C_1–1_H_h+3_ N_5_O_o–4_S_2_) at a mass difference of only 0.026 mDa.^16,17^ The challenge for the user is to decide which assignment is correct (in the absence of authentic standards or tandem MS data), particularly because replacement pairs do not indicate chemical relationships between molecules and are only theoretical solutions within the limits of instrumental accuracy and the considered set of elements. Without reliable evaluation and decision methods, MultiAs lead to the inclusion of false or improbable MFs and complications for biogeochemical data interpretation.^14,16^

The key challenge for MF assignment in highly complex mixtures is that the solutions for the Diophantine equation increase dramatically as molecular mass increases or heteroatoms or stable isotopes (e.g., ^13^C and ^34^S) are added.^14^ The inclusion of additional elements and their stable isotopes, such as necessary for the analysis of disinfection byproducts (^35^Cl and ^37^Cl), in mechanistic studies using stable isotope labeling (e.g., ^2^H and ^18^O), or for organo-metal complexes (e.g., Fe), leads to a wealth of new MultiAs, which makes the identification of correct assignments difficult.^18−22^

To address these issues, several empirical rules have been proposed for biogeochemical and related fields:

(1) A priori restriction of the formula assignment to important elements (C, H, O, N, and S). While this method achieves unambiguous formula assignment with high accuracy for low mass ranges, this is not always possible for the whole mass range (typically up to 1000 Da), and many peaks can remain unassigned.^14,23^ Another problem is that exclusion of certain elements can keep false assignments unrecognized in the data. For instance, sodium adducts of CHO-class MFs measured in negative mode electrospray ionization can be falsely assigned as CHOS-class MFs because Na is often not considered in studies using ESI(−).
(2) Building blocks, or homologous series, facilitate MF assignment algorithms. Due to the high complexity, mass spectra of NOM show remarkably regular patterns and MFs can be grouped into “molecular families” or “homologous series” and evaluated via Kendrick mass defect (KMD) analysis.^24,25^ Unequivocally assigned formulas at low masses (cf. (1)) are extended to higher mass range within homologous series. However, if the identification of the lightest member of a series is ambiguous or wrong, the whole series might be incorrect and false assignments cannot be excluded.^14^
(3) The presence/absence of stable isotope signals delivers an intrinsic chemical validation and is considered the “gold-standard” in formula assignments.^26^ In addition, the peak intensity of isotopologues, such as containing ^13^C, ^34^S, and ^37^Cl, provides information on the number of the major isotopes (^12^C, ^32^S, and ^35^Cl) in the parent molecule.^14,27^ However, the precision of the procedure strongly depends on signal intensity^14,24^ and is therefore not applicable for parent ions with low abundance and problematic for heteroatoms that only have isotopes with low natural abundance, such as ^15^N (0.4%).^28,29^ For elements that only have one natural stable isotope, e.g., ^31^P and ^19^F, the procedure cannot be applied at all.

Despite the integration of various rules into sophisticated software tools,^27,30−34^ numerous false assignments in MF data sets can still remain and require a posteriori judgment by experts.^16,17,35^ Hence, a universal and robust criterion is urgently needed to differentiate less reliable MF assignments from the most probable ones.^36^

Here, we propose the use of the median value of mass error distributions in millidalton (mDa) of the KMD series as a robust criterion for the recognition of false assignments in MultiAs (Figure S1). A workflow implementing this approach was developed to evaluate whole KMD series and to remove groups of false assignments. The workflow was applied to chlorine-containing and stable isotope (^2^H and ^18^O)-labeled compounds measured with UHRMS, and its performance was tested against published validation rules for NOM.

## Experimental Section

### Description of FT-ICR MS Data Sets and Experimental Procedures

To develop our workflow and test its applicability to resolve MultiAs in UHRMS data sets, we used five FT-ICR MS data sets (new data sets: *SRFA*, *SRFA_Na*, and *SRFA_CBZ_2H*; previously published data sets: *DW_Cl2* and *EfOM_Oz_18O*),^18,37^ all acquired with negative mode electrospray ionization on the same instrument (12 T SolariX XR, Bruker Daltonics Inc., Billerica, MA, USA) using direct infusion (DI) or liquid chromatography. (1) Suwannee River fulvic acid [SRFA III (3S101F) from the International Humic Substances Society] represents a complex organic matter mixture with elements and isotopes at their natural abundance and was measured with DI (*SRFA* data set). (2) SRFA was photoirradiated together with carbamazepine-*d*_10_ (Isotopic purity >95%, Toronto Research Chemicals, Toronto, CA)—a process, which has been shown to form covalent bonds between NOM molecules and carbamazepine–introducing deuterium (D, indicated also as ^2^H below) into NOM-MFs. This sample was measured with LC-FT-ICR MS. Chromatograms were divided into 1 min long segments, each of which were averaged into one mass spectrum and treated as DI.^38^ For one sample, 16 spectra were obtained (*SRFA_CBZ_2H* data set). (3) and (4) We also used previously published LC-FT-ICR MS data from drinking water (DW) disinfected with chlorine, introducing ^35^Cl and ^37^Cl at their natural abundance (*DW_Cl2* data set),^25^ as well as effluent organic matter (EfOM, from an effluent wastewater treatment plant) ozonated with heavy ozone (*EfOM_Oz_18O* data set).^9^ In the *DW_Cl2* data set, chlorine-containing MFs were identified as new peaks to which ^35^Cl-MFs could be assigned and that also have an accompanying ^37^Cl-isotopologue peak. In this case, the natural isotope abundance is helpful for the validation of the number of Cl atoms. Due to the chemical labeling with heavy isotopes, ^18^O (*EfOM_Oz_18O* data set) and ^2^H (*SRFA_CBZ_2H* data set) are introduced into the samples at high amounts, and natural isotope abundance cannot be directly used for the validation of ^18^O and ^2^H MFs. In the *EfOM_Oz_18O* data set, the isotope ratio of ^18^O, corresponding to ^16^O isotopologues produced as a result of the ozonation, was expected to be approximately 50% (from using ^18^O_3_). In contrast, for the *SRFA_CBZ_2H* data set, ^2^H is introduced by pure chemicals, which further undergo UV-degradation, introducing a variable number of ^2^H into NOM molecules and isotope ratios cannot be used for validation. (5) SRFA containing 5 mg/L sodium ions was measured with DI (*SRFA_Na* data set). Sodium adducts were observed and confirmed by comparison with the *SRFA* data set (i.e., without added sodium). Experimental details for all five data sets are described in the Supporting Information (cf. Supporting Information, sample description & Table S1). All spectra were internally calibrated in commercial software (DataAnalysis, version 6.0, Bruker Daltonics) with known CHO series (58 < *n* < 323). For LC analyses, each retention time segment was calibrated separately. After calibration, the root-mean-squared mass error (RMSE) was always <0.2 ppm and means of mass error (*M*_err_, in mDa) of calibrants were less than 0.010 mDa (mean: 0.006 ± 0.004 mDa; median: 0.001 ± 0.003 mDa) (Table S2 & Figure S3; Supporting Information: performance of internal calibrations). For all spectra, only peaks with a signal-to-noise ratio (S/N) larger than four were considered.

### Molecular Formula Assignment

MFs were assigned to all singly charged mass peaks in the range of *m*/*z* 147–1000 with an allowed relative mass error (RME) of 0.5 ppm. The only exception was data set *DW_Cl2*, for which only peaks in the range *m*/*z* 150–250 were measured and assigned with a tolerance of 1 ppm because it was obtained under continuous accumulation of selected ion (CASI) mode for better detection of chlorine MFs at low concentration levels in disinfected DW. In the assignment procedure, we allowed for all possible combinations of C, H, and O (C: 1–80, H: 1–198, O: 0–40), O/C (0–1.2), H/C (0.3–3), N/C (0–1.5), DBE (0–25), and DBE–O (−10–10). In addition, we used different setups for the number of heteroatoms or stable isotopes that we indicate in the following with the prefix CFC (chemical formula configuration), e.g., *CFC-N5S3* (Table S3). All detected MFs including isotopologues were used in the final data set and no other filters (e.g., based on isotope ratios) were applied before the mass error distribution assessment. MultiAs are those mass peaks, which have more than one MF assigned to them.

### Mass Error Distributions (in mDa)

MultiAs, that shared the same replacement pair were grouped, and the mass error (*M*_err_) calculated in mDa as the difference between the theoretical mass of the assigned MFs and the neutral measured mass (cf. Supporting Information, mass error in mDa and its distribution). By this, each mass peak that had MultiAs caused by a replacement pair had two *M*_err_ values. Note that we used *M*_err_ in mDa, not the more commonly used RME in ppm, because the mass differences of replacement pairs are constant but only in *M*_err_ in the (m)Da scale and not in the ppm scale (cf. Supporting Information: relative mass error and its distribution). Hence, groups of MultiAs result in a distribution of *M*_err_ values, and the true assignments are expected to follow normal distribution with a median of zero (providing sufficient mass calibration fitted by linear regression; cf. Supporting Information: mass error in mDa and its distribution). The *M*_err_ values of false assignments in the same MultiAs group are expected to also follow a normal distribution with homoscedasticity similar to their true-assignment counterparts but with nonzero median. The mass difference of the medians of the *M*_err_ distributions equals the mass difference between the members of the replacement pair. The member that has a *M*_err_ median closer to zero can be identified as the true assignments (Figure S1).

It should be noted that the RME may also reflect the difference between false and true positives (Figure S2), but the RME distribution is not suitable for the recognition of false assignments (Supporting Information, relative mass error and its distribution).

### Identification and Subsetting of MultiAs Based on KMD Values

The fixed mass difference between the members of replacement pairs also results in a fixed difference in KMD values. KMD values with a CH_2_ base were used to subdivide all formulas of a data set into homologous series. For homologous series that belong to a replacement pair, the median of the *M*_err_ distribution for each KMD was calculated.

### Validation of the New Approach with Existing Methods for True MF Assignment

The performance of our approach in identifying true assignments was validated in two ways:

First, for the *SRFA* data set, the performance of the approach was assessed by its ability to distinguish between true and false assignments in MultiAs caused by a replacement pair (H_3_N_5_S_2_/^12^C_5_^13^C_1_O_4_ with an extremely small mass difference of 0.026 mDa). Herzsprung et al. found that almost every (2209 out of 2213) “N5S2” MFs (^12^C_c–5_^13^C_1–1_H_h+3_N_5_O_o–4_S_2_) found in SRFA were in reality the ^13^C isotopologues of N- and S-free ^12^C mono-isotopologues (^12^C_c_^13^C_1_H_h_N_0_O_o_S_0_). This was confirmed by the exact Δ*m* of 1.003354 Da with mono-isotopologues and their reasonable δ^13^C distribution.^16^ According to that study, “N5S2” formulas in SRFA are false assignments caused by the false replacement of H_3_N_5_S_2_ residuals with ^12^C_5_^13^C_1_O_4_.^16^ Here, MultiAs in the *SRFA* data set only caused by this replacement pair (H_3_N_5_S_2_/^12^C_5_^13^C_1_O_4_) were used as a benchmark to test the ability of our approach to recognize and exclude false assignments. MFs with ^12^C_5_^13^C_1_O_4_ residuals and MFs with H_3_N_5_S_2_ residuals were hence regarded as condition positives (true assignments) and negatives (false assignments), respectively. MFs with ^12^C_5_^13^C_1_O_4_ residuals and MFs with H_3_N_5_S_2_ residuals recognized by our workflow could thus be classified as true positives (^12^C_5_^13^C_1_O_4_ retained by our workflow) and true negatives (H_3_N_5_S_2_ removed by our workflow), respectively. A confusion matrix was calculated accordingly (cf. Supporting Information: performance of workflow for data filtering).

Second, for the *DW_Cl2* data set, the performance of our approach was assessed by its ability to affirm ^35^Cl-containg MFs, which could be validated based on the robust isotope ratio filter as described elsewhere.^37^ Briefly, chlorine-containing MFs in MultiAs were first validated by the presence of accompanying ^37^Cl isotopologues and the expected mass peak intensity ratio of ^35^Cl vs ^37^Cl isotopologues. MFs validated by the isotope ratio filter were regarded as condition positives (true assignments), and associated false assignments in MultiAs were condition negatives. Next, MultiAs containing ^35^Cl in one replacement pair in the *DW_Cl2* data were inspected according to their *M*_err_ distribution by our workflow. The intersection between condition positives and the test outcome positives/negatives was again classified as true positives (retained by our workflow) and false negatives (removed by our workflow but independently validated as true by the isotope filter), respectively, and was used for the calculation of accuracy in the confusion matrix (cf. Supporting Information: *DW_Cl2* data set).

### Minimum Data Points for the Estimation of *M*_err_ Distribution

For a proper observation of median values of *M*_err_ distributions of subgroups, a minimum number of data points are required. Minimum data points were calculated by Lehr’s equation (cf. Supporting Information: performance of workflow for data filtering). For comparison of parameters between distributions in a more robust way, median values were consistently used in this study.

## Results and Discussion

### Recognition of False Assignments from Replacement Pairs Via *M*_err_ Distributions in mDa

Expectedly, more MFs were assigned to mass peaks in the *SRFA* data set when allowing for more N and S atoms in the assignment procedure (Table S4). However, this also resulted in an even larger increase of MultiAs, from 12% to 61%, when, for example, using *CFC-N5S3* instead of *CFC-N3S1* (Table S4). True and false assignments cannot be differentiated via the commonly used RME range of 1 ppm.^4^ In the *SRFA* data set assigned with *CFC-N5S3*, over 15,000 peaks were found having MultiAs, caused by more than 18 replacement pairs with a mass difference less than 1 mDa (equivalent to 1 ppm at *m*/*z* 1000). The 18 most frequent replacement pairs explained about 90% of all MultiAs (Table S5 and Figure S4). The most frequent replacement pair was H_8_N_2_S_3_/C_7_O_3_ having a mass difference of 0.217 mDa and accounting for 15% of all MultiAs. The smallest mass difference was identified for the replacement pair H_3_N_5_S_2_/^12^C_5_^13^C_1_O_4_ (0.026 mDa), which accounted for 10% of MultiAs in the *SRFA* data set. In contrast, when the *SRFA* data set was assigned with *CFC-N3S1*, only 2060 peaks had MultiAs, and the replacement pair ^13^C_1_H_1_N_3_O_4_/C_10_ with mass difference of 0.060 mDa explained about 84% of the MultiAs (Table S5).

A priori exclusion of elements or restricting the number of heteroatoms, as with *CFC-N3S1*, are thus feasible strategies to rule out over 85% of the false assignments caused by replacement pairs with more than 3 nitrogen and/or 1 sulfur atoms (Table S4). However, MultiAs caused by ^13^C_1_H_1_N_3_O_4_/C_10_ (0.060 mDa) and H_4_N_2_O_2_S/C_8_ (0.651 mDa) still remained and needed further evaluation (Table S5). Although the number of heteroatoms in the CFC could be further limited, this would leave most of the heteroatomic formula classes (CHNO, CHOS, and CHNOS) unseen in the data set, even though they occur in every environmental compartment and play important roles in ecosystems.^39−42^

**Figure 1:**
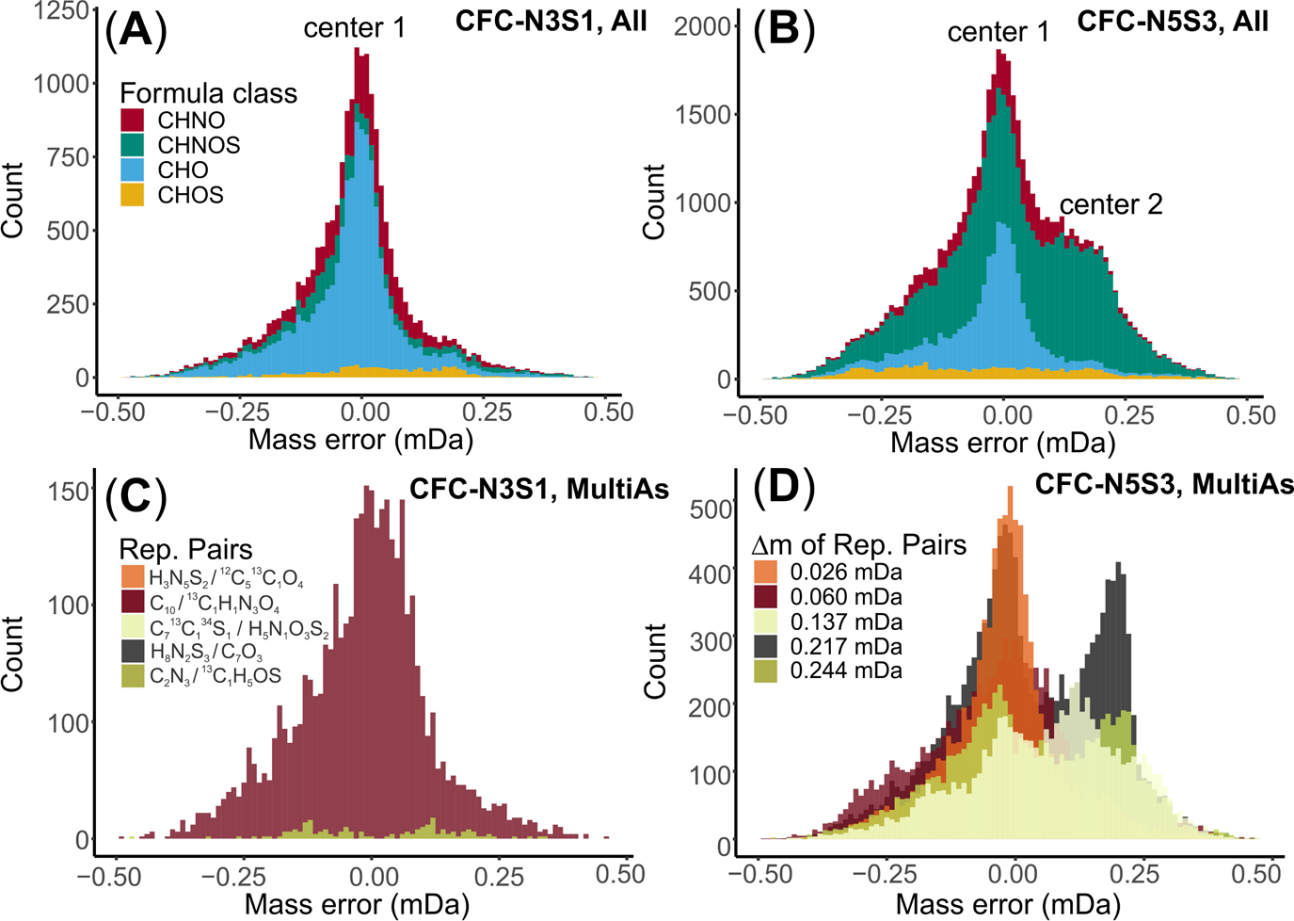
Mass error (M_err_) distribution of the *SRFA* data set assigned with different CFCs. (A) All formulas from *CFC-N3S1* in 4 main classes (*n* = 19,493), with one maximum close to zero; (B) all formulas from *CFC-N5S3* in 4 main classes (*n* = 50,720), showing two distinct maxima; (C) MultiAs caused by dominant replacement pairs in *CFC-N3S1* (*n* = 3740), and (D) MultiAs caused by dominant replacement pairs in *CFC-N5S3* (*n* = 44,723). Colors in C and D refer to replacement pairs involved in MultiAs and their corresponding mass differences. Note the different scaling of the *y*-axes.

*M*_err_ for all MFs in the *SRFA* data set assigned with *CFC-N3S1* displayed a normal distribution with one maximum (center) and 88% unambiguous assignments (Figure 1A). *M*_err_ of only the MultiAs were unimodally distributed and mainly caused by the replacement pair ^13^C_1_H_1_N_3_O_4_/C_10_ (0.060 mDa) and ^13^C_1_H_5_OS/C_2_N_3_ (0.244 mDa) (Figure 1C). When expanding the number of heteroatoms to *CFC-N5S3*, a bimodal distribution of *M*_err_ was observed considering all MFs; the second center mainly resulting from a large number of MultiAs from H_8_N_2_S_3_/C_7_O_3_ (0.217 mDa) (Figure 1B,D). MFs in such bimodal distributions with centers clearly different from zero could be easily recognized and labeled as false assignments.

**Figure 2:**
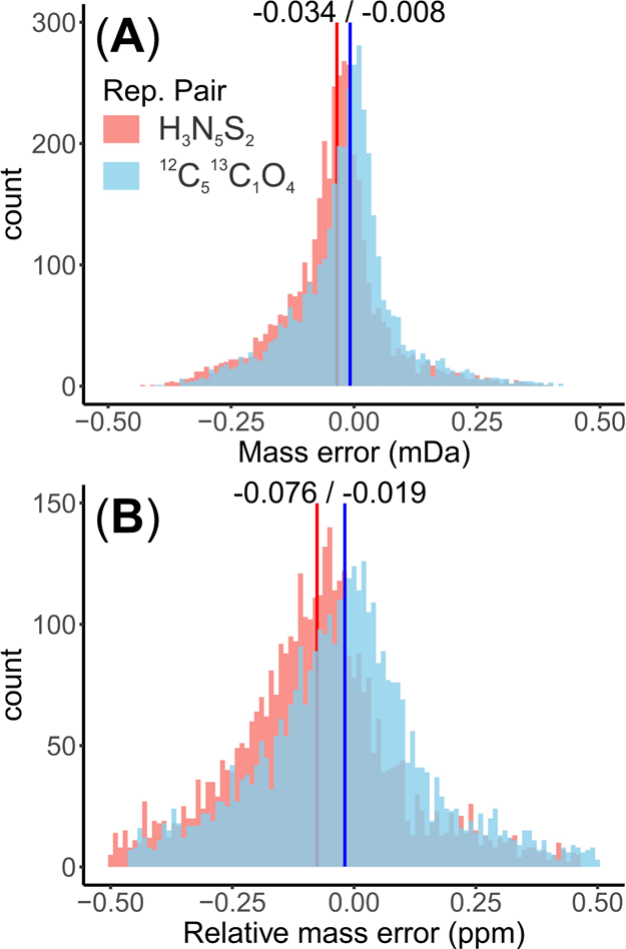
Example MultiAs and its replacement pair in the *SRFA* data set (H_3_N_5_S_2_ vs ^12^C_5_^13^C_1_O_4_, 0.026 mDa mass difference, *n* = 7640): (A) overlapped mass error (M_err_) distribution of MultiAs from replacement pair H_3_N_5_S_2_ vs ^12^C_5_^13^C_1_O_4_ [with median values of −0.034 mDa (“N5S2” MF) and −0.008 mDa (“^13^CHO” MF) for false and true assignments]; (B) overlapped RME distribution of same MultiAs [with median values of −0.076 ppm (“N5S2” MF) and −0.019 ppm (“^13^CHO” MF) for false and true assignments].

The applicability of the approach can be demonstrated by inspecting the *M*_err_ distribution of MultiAs from the prominent replacement pair H_3_N_5_S_2_ vs ^12^C_5_^13^C_1_O_4_ with mass difference of just 0.026 mDa (Table S5). While the bimodal distribution considering all MFs from this replacement pair is hardly visible (Figure 2), the H_3_N_5_S_2_ MFs can still be recognized as false assignments by their nonzero median of −0.034 mDa (Figure S5). Although this replacement pair can be evaluated based on isotopic and chemical evidence,^16^ our data demonstrate that another criterion (namely *M*_err_ distributions in mDa) can be used to differentiate true and false assignments (see below). Notably, the *M*_err_ distribution criterion is independent of isotopic evidence or structural constraints of NOM molecules, opening the possibility for a generic approach that includes also MFs with low abundance.

This generic character of the *M*_err_ distribution approach is demonstrated for CHOS formulas that are, in fact, Na adducts of oxygen-rich CHO formulas. Such Na adducts can form in samples that are not sufficiently desalted or contain large amounts of oxygen-rich molecules and can be detected by ESI(−) mode (Figure S7). However, Na is usually not considered a potential element for formula assignment for measurements obtained with ESI(−). When Na is considered, initial unequivocal CHOS MFs became ambiguous and occurred as MultiAs (cf. Supporting Information: *SRFA_Na* data set). Here, 328 CHOS formulas in the *SRFA_Na* data set were found to be Na adducts of CHO molecules (CHO_Na) when applying *CFC-N5S3-Na*. These MultiAs were caused by the replacement of HO_5_Na with C_6_S with a mass difference of 0.096 mDa (Figure S8). All MFs had a S/N ratio <100, preventing the use of ^34^S-isotopologues for validation (natural abundance of ^34^S: 4.2%). These false CHOS formulas have a much lower H/C and O/C ratio than their CHO counterparts and appear in the lower left quadrant of the van Krevelen diagram (Figure S9), making them a biogeochemically interesting (“black sulfur”) group of molecules, with, however, presumably low ionization efficiency in ESI(−). Our results indicate that such CHOS MF should be taken with great caution and that checking the *M*_err_ distribution of CHOS formulas is highly recommended to limit these false assignments in NOM data.

Overall, distinct modes in *M*_err_ distributions of the full data sets are not always accessible by visual inspection (Figure 1), especially when MultiAs are caused by the replacement pairs with mass difference less than 0.1 mDa, e.g., H_3_N_5_S_2_/^12^C_5_^13^C_1_O_4_ and ^13^C_1_H_1_N_3_O_4_/C_10_ (Figures 1 and 2). Therefore, an independent, reliable method is needed to recognize replacement pairs from MultiAs and to distinguish true (i.e., most likely) and false (i.e., less likely) assignments.

Further, although *M*_err_ distributions differ between true and false assignments among MultiAs, grouping all MFs together that contain the same replacement pair (as in the case of H_3_N_5_S_2_/^12^C_5_^13^C_1_O_4_ and HO_5_Na/C_6_S; see above) may not always be appropriate. In case MFs within a group of one replacement pair consist of both true and false assignments, the *M*_err_ distribution might still occur as bimodal and using the medians of the *M*_err_ distribution might bias the validation. For instance, evaluation of MultiAs caused by replacement pair O_1_P_1_/C_1_^35^Cl_1_ with mass difference of 0.176 mDa may result in all CHOCl MFs regarded as true assignments due to their better agreement of ppm error distribution with calibrants (Figure S10A).^32^ However, a bimodal *M*_err_ distribution in mDa of CHOCl MFs from the *DW_Cl2* data set was clearly observed (Figure S10B), suggesting coexistence of true and false assignments for both members of the replacement pair, i.e., false O_1_P_1_ MFs (here: true C_1_^35^Cl_1_) coexist with true O_1_P_1_ MFs in the same data set (Figure S10C), complicating the procedure of MF validation.

Hence, MultiAs in the whole data should be subset in proper ways so that false assignments in subgroups can be recognized with higher confidence by their nonzero medians of *M*_err_ distribution (Figure S1). The procedure of subsetting will be presented in the following sections.

### Automatic Recognition of False Assignments by KMD-Based MF Grouping

#### Description of the Workflow

Briefly, the input consists of the mass spectrometry information (measured and theoretical formula mass, calculated *M*_err_, formula class, and the KMD in the CH_2_ scale), after which the whole data set would be sliced into subgroups for the examination of *M*_err_ distribution. MFs were subsetted in groups that shared the same KMD values and replacement pairs. Then, the medians of *M*_err_ distributions were calculated for each subgroup. Subgroups with nonzero medians in MultiAs were considered as false assignments and discarded entirely, while subgroups with medians of zero were kept as true assignments.

The entire workflow was implemented as an R script available for download from https://git.ufz.de/lambda-miner/defender. The workflow is capable to validate >100 k MF assignments in less than 30 s on a standard Windows laptop computer (Table S20).

#### Benefit of KMD-Based Subsetting for Complex Samples

Recently, Jennings et al. reported the replacement of ^18^ON_2_ with CH_2_O_2_ to be the main culprit to MultiAs in EfOM samples ozonated with heavy oxygen.^18^ Accordingly, in the *EfOM_Oz_18O* data set, 6% of all mass peaks assigned (*n* = 13,900) with *CFC-N5S3-18O* had MultiAs remaining after limiting RME to ±0.2 ppm and ^13^C and ^34^S isotopologues evaluation, 94% of which were caused by the replacement pair ^18^ON_2_/CH_2_O_2_ with mass difference of 0.172 mDa (Tables S7 and S8). A bimodal *M*_err_ distribution was observed not only for all MultiAs (Figure 4A) but also for each replacement pair group (Figure 4B). This is in contrast to the H_3_N_5_S_2_/^12^C_5_^13^C_1_O_4_ replacement pair (Figure 2), indicating an unresolved mixture of true and false assignments within each group of the replacement pair ^18^ON_2_/CH_2_O_2_ and that grouping the data solely by the same replacement pair, as in the case of the H_3_N_5_S_2_/^12^C_5_^13^C_1_O_4_, thus seem not robust enough for a fully automated data filtering workflow.

**Figure 3:**
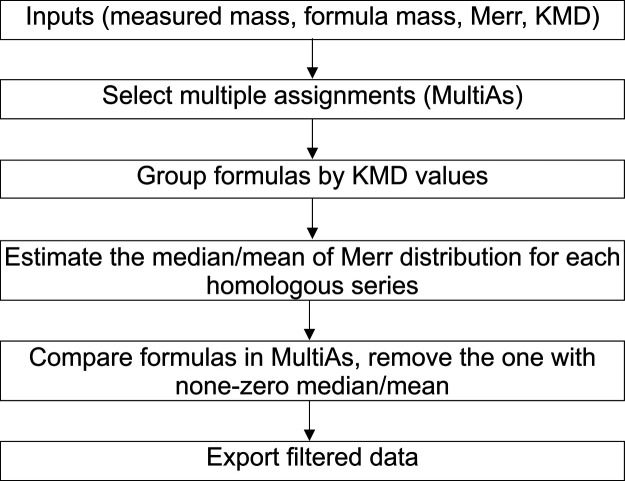
Workflow for recognition and screening of false assignments among MultiAs data via the mass error (*M*_err_) distribution in mDa.

**Figure 4:**
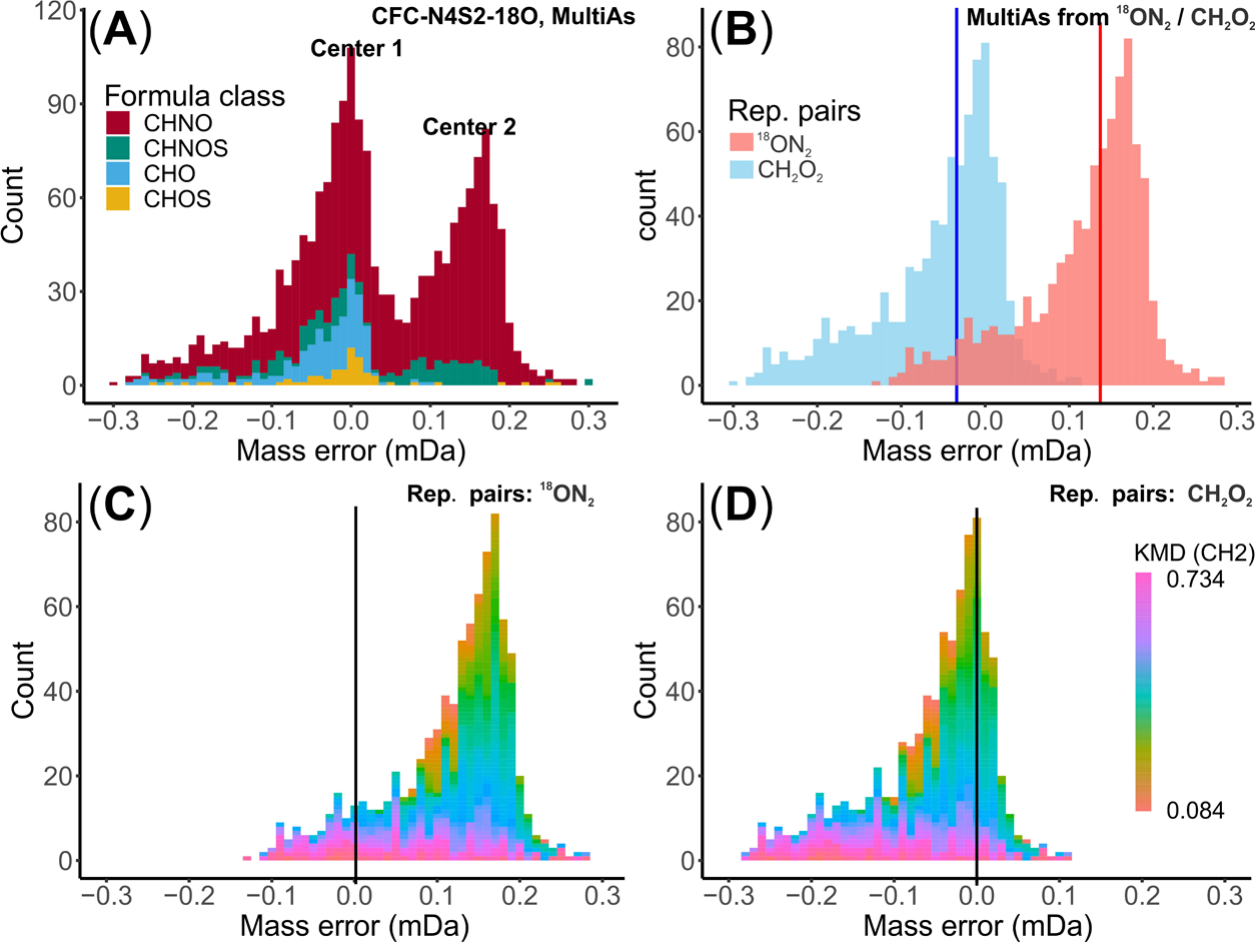
Mass error (*M*_err_) distribution of remaining MultiAs in the *EfOM_Oz_18O* data set: (A) *M*_err_ distribution of all MultiAs with two maxima (centers) (*n* = 1810); (B) *M*_err_ distributions of ^18^ON_2_ MFs and CH_2_O_2_ MFs (corresponding to median values of 0.137 mDa shown as a red line and −0.034 mDa shown as a blue line; *n* = 1696); (C) *M*_err_ distributions of ^18^ON_2_, and (D) *M*_err_ distributions of CH_2_O_2_ MFs with colors referring to CH_2_-based KMD values.

Inspection of KMD values within *M*_err_ distribution of each group revealed the presence of further modes, representing subgroups of chemically distinct molecules (Figure 4C,D). For MFs with CH_2_O_2_ residuals, most of the CH_2_-based homologous groups were true assignments and had *M*_err_ distributions around 0, but some were false assignments with *M*_err_ distributions near −0.170 mDa. For MFs with ^18^ON_2_, despite false assignments with the *M*_err_ center at 0.170 mDa, there were still true assignments with *M*_err_ around 0. This indicates that globally excluding MFs only by their specific replacement pairs could yield erroneous results. As a robust and versatile criterion for MF grouping in MultiAs, we thus propose to evaluate *M*_err_ distributions in KMD homologous series. Accordingly, MFs within the whole set of MultiAs in a data set will be grouped if they share the same KMD value and formula residual after which the median of the *M*_err_ for each subgroup will be considered for validation (Figure 3). In the *EfOM_Oz_18O* data set, this resulted in a rejection of 848 MFs (582 ^18^ON_2_ and 266 CH_2_O_2_), whereas 848 MFs (266 ^18^ON_2_ and 582 CH_2_O_2_) were retained as valid (Table S9).

Of note, other MF subsettings via formula class identifiers (e.g., nominal mass series z* or family score) are less suitable than the KMD as they either result in too small or large but unspecific groups.^43,44^

#### Implementation of the Workflow and Performance for Automatic Data Filtering

MultiAs in the *SRFA* data set assigned with *CFC-N5S3* were filtered by the workflow described above (cf. Supporting Information: *SRFA* data set). Out of 51,476 MFs before the filtering of MultiAs, 25,109 MFs were kept and 26,367 MFs were rejected (Tables S4 and S6), resulting in a reduction of the MultiAs rate from 61% (15148 peaks involved) to 1.5% (375 peaks involved) (Tables S4 and S6 and Figure S6). An overall normal distribution of all filtered MFs could be estimated with a median of −0.001 mDa and a standard deviation of 0.124 mDa (Table S11).

Before filtering, the exchange of ^12^C_5_^13^C_1_O_4_ with H_3_N_5_S_2_ caused MultiAs of 7640 formulas, corresponding to 3820 peaks (Table S10). The automatic data filtration based on KMD subgroups resulted in the rejection of 4821 MFs as false assignments (H_3_N_5_S_2_: 3245 and ^12^C_5_^13^C_1_O_4_: 1576) and the inclusion of 2819 MFs as true assignments (H_3_N_5_S_2_: 575 and ^12^C_5_^13^C_1_O_4_: 2244), corresponding to an accuracy of 72% (Table S10). Here, instead of globally rejecting H_3_N_5_S_2_ MFs (cf. Figure 2), the exclusion of H_3_N_5_S_2_ MFs were performed within smaller KMD series.

The accuracy of this approach (i.e., distinguishing between median values of two distributions) depends on two factors: the mass difference of the replacement pair and the width of the *M*_err_ distribution (e.g., expressed as its standard deviation). According to the statistical power, in case of a fixed standard deviation (which depends on the achievable mass accuracy), the sample size (i.e., number of MFs in the KMD subgroup) needs to increase for decreasing differences in the medians.^45^ For example, for the case of H_3_N_5_S_2_/^12^C_5_^13^C_1_O_4_ in the *SRFA* data set, the standard deviation of *M*_err_ was 0.105 mDa, and hence a minimum 130 data points were expected to properly estimate medians at 0.026 mDa mass difference (α = 0.05, Table S11), which was the smallest mass difference in the *SRFA* data set (Table S5). The required sample size then decreases with an increasing difference in the medians of replacement pairs (Figure S11A). KMD series in the *SRFA* data set had sample sizes below 50, with averages ranging from 2 to 15 depending on the considered replacement pairs. For replacement pairs with mass difference above 0.150 mDa (e.g., C_3_H_7_S_3_/^13^C_1_NO_7_ with a mass difference of 0.158 mDa), most of the sample sizes were larger than required for estimation of medians of *M*_err_ distribution with a standard deviation of 0.105 mDa.

Another way to improve the accuracy of the approach is to reduce the standard deviation of the *M*_err_ distributions, which represents the mass and calibration accuracy. The minimum sample sizes for estimation of medians decrease with the standard deviation dropping from 0.105 to 0.025 mDa (Figure S11B). To improve mass accuracy, FT-ICR-MS with higher magnetic field strength, e.g., 15 to 21 T or quadrupolar detection may be employed,^1^ both of which would require costly instrument upgrades. Alternatively, the mass accuracy can also be improved by better mass calibration. For example, walking calibration has been shown to be suitable for samples with more heteroatoms and can reduce the RMSE by as much as 3-fold.^46^ Similarly, absorption mode spectral processing (AMP) can also improve data quality at low extra costs.^9^ Assignment windows with ±0.25 ppm mass accuracy have been reported to be feasible for NOM samples via AMP on 12 T FT-ICR MS.^13^

### Applications to Data Sets with Stable Isotope Labeling

#### Filtration of MultiAs in the ^35^Cl/^37^Cl Related Data Set

The introduction of chlorine makes formula assignments more challenging, due to more MultiAs, when several ^35^Cl and ^37^Cl are included, e.g., with *CFC-N*_*2*_*S*_*1*_^*35*^*Cl*_*3*_^*37*^*Cl*_*3*_.^37^ Here, we focus on MultiAs caused by chlorine-containing MFs in replacement pairs when other heteroatoms are tightly limited. Before data filtration, 6267 MFs were assigned in the *DW_Cl_2_* data set to 3780 peaks, of which 39% were involved in MultiAs (Table S12). Our automatic data filtering based on KMD groups resulted in the rejection of 2487 MFs and the inclusion of 3780 MFs, decreasing the MultiAs rate to zero (Table S14). In total, 969 ^35^Cl MFs were considered valid according to their near-zero median *M*_err_ values (Table S15). Out of those, 395 MFs with ^35^Cl had an accompanying ^37^Cl isotopologue MF.

In contrast, only 124 ^35^Cl/^37^Cl MF pairs passed the exclusive isotope ratio filtering and were regarded as valid formulas (Table S16). Out of those, 98 ^35^Cl MFs from isotope ratio filtered data were also validated by the new workflow using *M*_err_ distributions, resulting in an accuracy of 80%. Notably, over 8-fold more ^35^Cl MFs could be validated by the inspection of *M*_err_ distributions as compared with only using isotope ratios. In fact, only one-third of the ^35^Cl MFs had accompanying ^37^Cl isotopologues (Table S15) with S/N ratios >5 (Figure S12A), while two-third of the ^35^Cl MFs had low S/N around 4 resulting in undetected ^37^Cl isotopologues (Figure S12B). Moreover, ions which were close in the cyclotron radius in the ICR cell may interfere with each other and cause a bias in relative abundances and will lead to a biased isotope ratio calculated from peak intensities compared to the expected intensity ratio from the natural abundance (Figure S13).^14,47^

The replacement pair ^35^ClO_3_/CH_2_^37^ClS (0.027 mDa) was responsible for about 32% of MultiAs (Table S13 and Figure S14). These formulas suggested over 400 unequivocal chlorine-containing MFs because both replacement pairs contained either ^35^Cl or ^37^Cl, the latter requiring the presence of ^35^Cl mono-isotopologues. According to the *M*_err_ filter, 362 of the ^35^ClO_3_ MFs in the replacement pair are true assignments, and 270 of them still have ^37^Cl isotopologues after filtering (Table S17). This suggests the applicability of *M*_err_ distribution for the validation of the *DW_Cl_2_* data set because those ^35^Cl mono-isotopologues, which have no ^37^Cl isotopologues or with biased ^35^Cl/^37^Cl intensity ratios can still be verified in this manner. This expands the analysis window for chlorine-containing compounds in nontargeted studies of disinfection byproduct formation.^37^

#### Filtration of MultiAs in the ^2^H Related Data Set

As discussed above, MultiAs can be validated in a robust manner based on their *M*_err_ distribution, even if no isotope intensity patterns can be used for validation. Here, deuterium (^2^H or D) was introduced into NOM molecules via photoinduced covalent bond formation with a stable isotope-labeled chemical.

**Figure 5:**
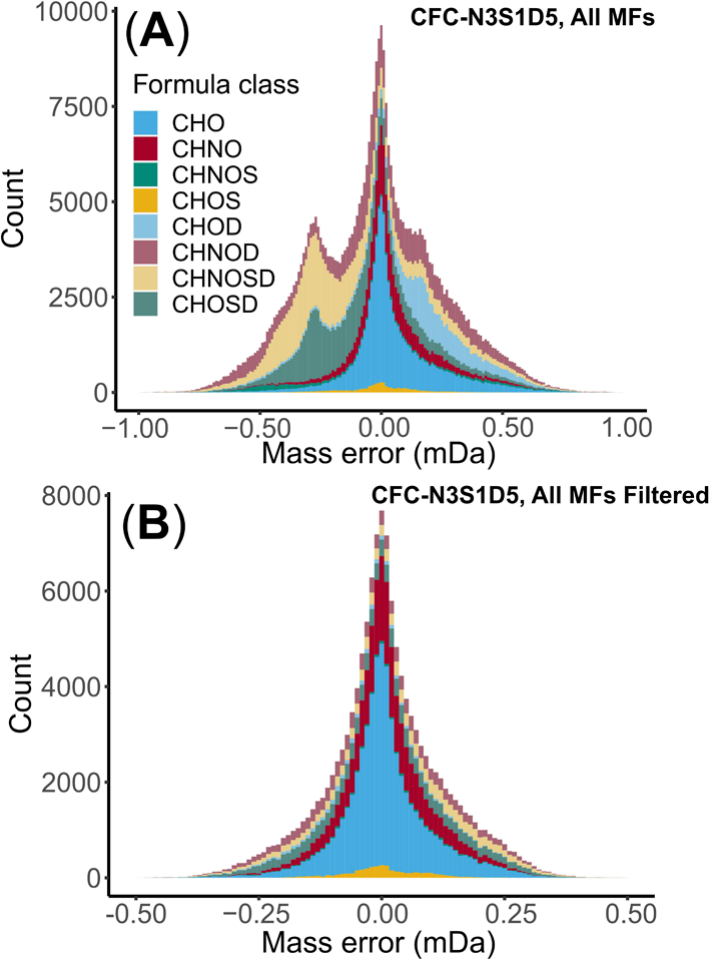
*M*_err_ distribution in the *SRFA_CBZ_2H* data set in mDa: (A) all formulas in 8 main classes (including D) before filtration (*n* = 383,420); (B) formulas in 8 main classes (including D) filtered by the *M*_err_ inspection subset from the KMD-CH_2_ class (*n* = 130,730).

Before data filtration, the overall data showed multimodal distribution in *M*_err_, with around 70% of the peaks having MultiAs (Figure 5A and Tables S18 and S19). Via the *M*_err_ filters, 316,202 formulas were removed from a total of 447,416 MFs, and 131,214 valid formulas were validated. The *M*_err_ of all MFs were normally distributed after the data filtration, indicating a low proportion of remaining MultiAs (Figure 5B).

Deuterium atoms contributed to most of the MultiAs, 43,124 MFs with ^2^H were retained from initially assigned 302,262 MFs containing ^2^H. The multimodal *M*_err_ distribution in MultiAs was also replaced by a unimodal distribution after the automatic filter workflow (Figure S15), indicating successful removal of false assignments.

## Conclusions

Up to now, the configuration of MF assignments for complex mixtures measured with UHRMS has been limited by the capability to extract valid formulas from many chemically feasible possibilities. If only small portions of N, P, and S are expected in samples, MultiAs may be regulated by strict element limits. However, many peaks in FT-ICR mass spectra may remain unassigned, potentially leaving biogeochemical information unconsidered. Leveraging the full potential of FT-ICR MS thus requires inclusion of more heteroatoms, metals, and stable isotopes at the cost of increasing MultiAs also for previously unequivocal MFs. Many MultiAs are caused by replacement pairs within the empirical mass error threshold and challenge even the most accurate mass spectrometers.

We could demonstrate that a generic criterion for recognition of false assignments using the statistical distribution of the mass error in mDa within a homologous (KMD) series is suitable for formula validation. Instead of RME comparisons and case-by-case evaluation, we utilize the fact that false assignments show nonzero medians in groupwise mass error distributions and can be excluded simultaneously. This accelerates robust formula assignment, particularly for peaks with low S/N and decreases the reliance on isotope intensity patterns for formula validation. Our approach can be used to validate MFs in samples with complex CFCs including N, P, and F and extends the applicability of FT-ICR MS in characterization of NOM, e.g., for organic nitrogen and organic phosphorus, which are key components for global elemental cycles.^39^

Formulas from experiments applying stable isotope labeling, such as ^18^O and D, for which natural isotope abundance cannot be used, can be verified without presumptions on chemical structures. Now, the fate of organic pollutants in natural waters and the formation of bound residues in different environmental compartments can be elucidated in detail with different options of isotope labeling.^48^ Likewise, more structural information for organic matter fractions become accessible via tagging functional groups with stable isotopes, e.g., via CD_3_OD and NaBD_4_ reactions.^22^

Based on this approach, a workflow was developed for automatic data filtration based on KMD homologous series and inspection of medians, achieving 72% accuracy for MultiAs with a mass difference as low as 0.026 mDa. Further improving mass and calibration accuracy will allow for a better estimation of medians especially for smaller sample sizes, eventually also facilitating MF assignment in less complex mixtures, such as metabolomics.

## Data Availability

All data sets used and the R script for implementing MultiAs filtering (“https://git.ufz.de/lambda-miner/defender.git”). Duration reported when running R codes for provided data is given in Table S20.

## Supporting Information Available

Additional information regarding sample description, internal calibration performance, CFC for MF assignments, schema for mass error distribution, MultiAs and replacement pairs, mass spectrum of sodium adducts, sample size, number of MFs and MultiAs before and after filtering, performance of the workflow, and performance of chlorine isotope filters (PDF)
