# Supplementary material for: Detection and Exclusion of False-Positive Molecular Formula Assignments via Mass Error Distributions in UHR Mass Spectra of Natural Organic Matter
Source: Anal Chem. 2024 Jun 13;96(25):10210–8. doi: 10.1021/acs.analchem.4c00489 (PMC11209664; doi:10.1021/acs.analchem.4c00489)
Supplement: Supplementary file 1 — ac4c00489_si_001.pdf [file ac4c00489_si_001.pdf]

Detection and Exclusion of False-Positive Molecular Formula Assignments via Mass Error (mDa) Distributions in UHR mass Spectra of Natural Organic Matter.

Shuxian Gao[1], Elaine K. Jennings[1], Limei Han[1], Boris P. Koch[2,3],

Peter Herzsprung[4], and Oliver J. Lechtenfeld[1,5,*]

[1] Helmholtz Centre for Environmental Research - UFZ, Department Environmental Analytical Chemistry, Research Group BioGeoOmics, Permoserstr. 15, Leipzig D-04318, Germany

[2] Helmholtz Centre for Polar and Marine Research - AWI, Department of Biosciences, Ecological Chemistry, Am Handelshafen 12, Bremerhaven D-27570, Germany

[3] University of Applied Sciences, An der Karlstadt 8, 27568 Bremerhaven, Germany

[4] Helmholtz Centre for Environmental Research - UFZ, Department Lake Research, Brückstr. 3a, Magdeburg D-39114, Germany

[5] Helmholtz Centre for Environmental Research - UFZ, ProVIS - Centre for Chemical Microscopy, Permoserstr. 15, Leipzig D-04318, Germany

* **Corresponding author**:

Oliver J. Lechtenfeld

E-mail: oliver.lechtenfeld@ufz.de

Table of Contents

Mass error (M_{err}) in mDa and its distribution 4

Figure S1. Schema for mass errors (mDa) distribution in multiple assignment caused by specific replacement pair with varying mass difference. 5

Relative mass error (RME) and its distribution 6

Figure S2. Schema for distribution of relative mass errors. 6

Sample description 7

Table S1. Description of samples and data acquisition. 7

Performance of internal calibrations and robustness of median/mean value of M_{err} distribution. 9

Table S2. Performance of internal calibrations. 10

Figure S3. Performances of internal calibrations from SRFA_CBZ_2H datase. 10

Molecular formula assignments 11

***SRFA* dataset** 13

Table S4. Total number of formula assignments in SRFA dataset before automatic filtration. 13

Table S5. Main replacement pairs that cause multiple assignments (MultiAs) in SRFA dataset. 13

Figure S4. Frequency of replacement pairs in MultiAs observed in SRFA dataset with 2 different CFC. 14

Figure S5. Example multiple assignment and its replacement pair in SRFA dataset. 14

Table S6. Total number of formula assignments in SRFA dataset after automatic filtration. 14

Figure S6. M_{err} distribution of SRFA dataset. 15

***SRFA_Na* Dataset** 16

Figure S7. Expanded section from a full scan mass spectrum showing Na^+ adducts in SRFA_Na. 16

Figure S8. Mass error distribution of MultiAs caused by Na^+ adducts in SRFA_Na dataset. 17

Figure S9. van Krevelen plot of MultiAs caused by replacement pair of C_6S (CHOS molecular formula (MF)) vs HO_5Na (CHO_Na MF) in SRFA_Na dataset. 17

Multiple assignments caused by O_1P_1 / $C_1{}^{35}Cl_1$ 18

Figure S10. Mass error distribution of molecular formulas (MFs) related to O_1P_1 / $C_1{}^{35}Cl_1$. 18

***EfOM_Oz_18O* dataset** 19

Table S7. Total number of formula assignments in EfOM_Oz_18O dataset before automatic filtration. 19

Table S8. Main replacement pairs that cause multiple assignments (MultiAs) in EfOM_Oz_18O. 19

Table S9. Total number of formula assignments in EfOM_Oz_18O dataset after workflow filtration. 19

Performance of workflow for data filtering 20

Table S10. Performance of automatic data filtering algorithm for SRFA dataset. 20

Table S11. Gaussian distribution fitting of M_{err} in SRFA dataset. 20

Figure S11. Sample size needed for proper estimation of M_{err} distribution of different replacement pairs and number of KMD series in the SRFA dataset (A) and (B) sample size estimation with different SD (according to instrumental mass accuracy). 21

DW_Cl2 dataset **22**

Table S12. Total number of formula assignments in DW_Cl2 dataset before automatic filtration. 22

Table S13. Dominant replacement pairs that cause multiple assignments (MultiAs) in DW_Cl2 dataset. 22

Table S14. Total number of formula assignments in DW_Cl2 dataset after automatic filtration. 22

Table S15. Total number of Chlorine formula assignments in DW_Cl2 dataset before and after filtration. 22

Table S16. Performance of automatic data filtering algorithm for DW_Cl2 dataset. 23

Table S17. MultiAs caused by $CH_2{}^{37}ClS$ and ClO3 in DW_Cl2 dataset during Merr validation. 23

Figure S12. S/N distributions of ^{35}Cl formulas before data filtering (plotted with bin size of 1). 23

Figure S13. Initial ^{35}Cl/ ^{37}Cl isotopologue intensity ratio:. 24

Figure S14. Mass error distribution of ^{37}Cl dataset ($CH_2{}^{37}ClS$ and ClO_3, 0.027 mDa). 24

***SRFA_CBZ_2H* dataset** **25**

Table S18. Dominant replacement pairs that cause multiple assignments (MultiAs) in one measurement in SRFA_CBZ_2H dataset (measurement: CBZ_D10_SRFA_pho_1_17_18min). 25

Table S19. Total number of formula assignments in SRFA_CBZ_2H dataset before and after filtration. 25

Figure S15. Mass error distribution of ^{2}H formulas. 25

Table S20. Duration reported when running R script snippet for different data inputs. Results were tested on laptop with CPU of Intel-i7, and SSD of 512 GB, R version 4.2.1. 26

Mass error (M_{err}) in mDa and its distribution

FT-ICR mass spectrometry principle can be written below by the mapping function F from mass-to-charge ratio (*m/z*) and other relevant physical quantities p_i, 1<i≤m (ion abundance etc.) to the corresponding ion motion frequencies *f* for a given mass analyzer:

$$f = F(m/z, p_1, \ldots, p_m) \qquad (1)$$

Then, the eq.1 can be solved for observed $(m/z)_{obs}$:

$$m/z = M_0(f, p_1, \ldots p_m) \qquad (2)$$

However, due to various effects, e.g. ion abundance and uneven electric fields, eq 2 is not even applicable until proper sufficient mass accuracy is provided by mass calibration functions.

To reduce the systematic errors in the measurements, the mass calibration function $M_{cal}(f, p_1, \ldots, p_m)$ could be fitted by *f* with corresponding theoretical m/z of internal calibrants ($(m/z)_{int}$):

$$(m/z)_{int} = M_{cal}(f, p_1, \ldots p_m) \qquad (3)$$

Then the $(m/z)_{corr}$ with enough mass accuracy could be obtained by applying M_{cal} to all other (m/z)obs.

$$(m/z)_{corr} = M_{cal}(f, p_1, \ldots p_m) \qquad (4)$$

When doing linear calibration, the parameters that need to be decided in eq 3 might be fitted by multivariate linear regression (LS) approach. LS here aims to minimize the root-mean-squared mass error (RMSE), $S = \sqrt{\sum_{i=1}^{N} {r_i}^2}$, where $r_i = y_i - \hat{y}$ is the residual for the *i*th data point and $\hat{y}$ is the fitted response value. In this case, y_i here is measured m/z, and fitted response value is theorical m/z. In practical, the residual will also be expressed as mass error (M_{err}) in mDa, which is the difference of measured mass and theoretical formula mass, by adding/removing a proton to m/z, considering the charge state of ion is ±1.

$$r_i = mass\ error\ (\Delta m_i)$$

$$= m/z_{theo.} - m/z_{meas.} \text{ in Da}$$

$$= (m/z_{theo.} - m/z_{meas.}) \times 10^3 \text{ in mDa.} \quad (5)$$

For LS, one of the assumptions is that the errors of the response variables are uncorrelated with each other, i.e. independence of errors, which means the residuals, or M_{err}, have normal distribution $\varepsilon \mid X \sim N(0, \sigma^2)$. Therefore, M_{err} shall follow normal distribution with mean value of 0.

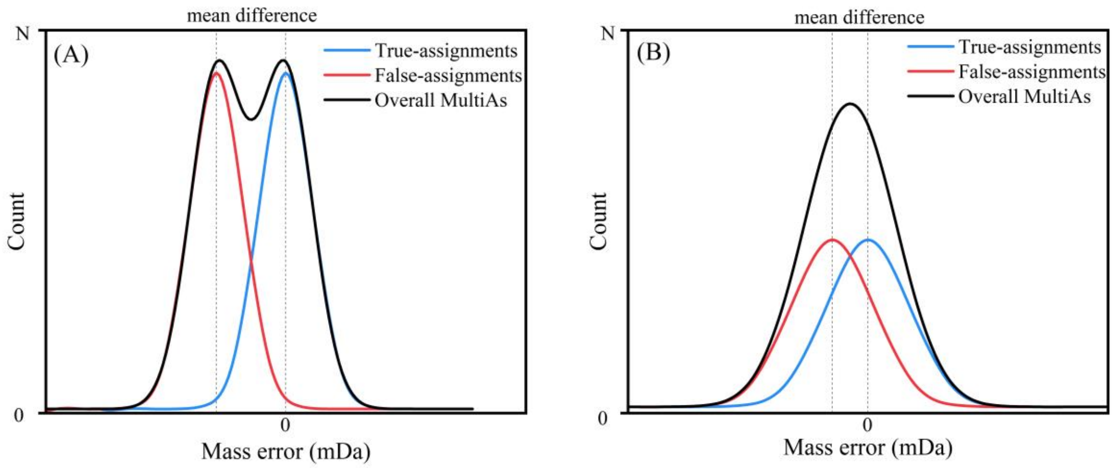

Figure S1. *Schema for mass errors (mDa) distribution in a multiple assignment caused by specific replacement pair with varying mass difference: (A) Mass difference of replacement pair is larger than twice the standard deviation of the M_{err} distribution of true-assignments resulting in a bimodal distribution with two local maxima; (B) Mass difference of replacement pair is less than twice the standard deviation of the M_{err} distribution of true-assignments resulting in a unimodal distribution with one non-zero center. Recognition of false-assignments is possible, if the underlying distributions can be recognized.*

Relative mass error (RME) and its distribution

It's should be noted that relative mass error in ppm (eq. 6) doesn't follow normal distribution as mass errors in mDa have.

$$\text{mass error } (\Delta m_i) = \frac{(m/z_{theo.} - m/z_{meas.})}{m/z_{theo.}} \times 10^6 \text{ in ppm (parts per million)} \quad (6)$$

Assume the mass follows normal distribution as well, i.e. m | X ~ $N(\mu, \sigma_m^2)$. Then the ratio of these two normal distribution $F_z = \frac{(\varepsilon \mid X)}{(m \mid X)}$ shall not be normal distributed. F_z has no finite moments and is heavy tailed, which shape can be bimodal, asymmetric, symmetric, and even close to a normal distribution, depending largely on the values of the coefficient of variation of m | X.[1] F_z in this case will be centered at $\beta = E(\varepsilon)/E(m)$. **If E(ε) is 0, then β equals 0, otherwise β is a non-zero but variable value depend on E(*m*).** Also, when F_z is estimated as Cauchy distribution, the mean values might not be obtained and the median values should be used. **Considering the availability of mean value and the large leverages of outlier biases, especially in small data groups, median value seems to be more robust for practical usage.**

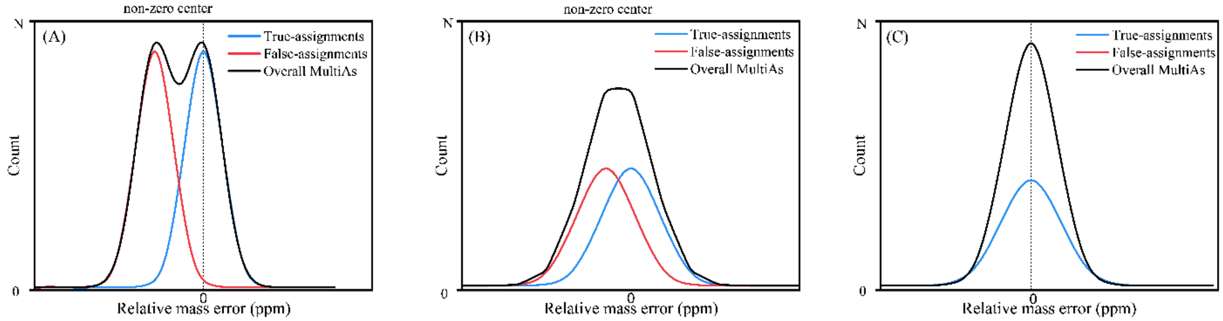

Figure S2. *Schema for distribution of relative mass errors. (A) bimodal distribution with 2 peaks; (B) unimodal with one non-zero center and (C) unimodal with one zero center stacked from complete overlap of true- and false assignments.*

Additional Reference:

(1) Díaz-Francés, E.; Rubio, F. J. On the Existence of a Normal Approximation to the Distribution of the Ratio of Two Independent Normal Random Variables. *Stat Papers* **2013**, *54* (2), 309–323. https://doi.org/10.1007/s00362-012-0429-2.

Sample description

Samples in this study are from different sources with treatment processes applied, and the FT-ICR spectra are obtained with different measurement modes, direct infusion (DI) or hyphenated with LC. Every sample is diluted to 10 mg/L DOC with ultrapure water for FT-ICR-MS analysis.

Table S1. *Description of samples and data acquisition.*

Dataset	Sample ID	Treatment process	Concentration	Data acquisition	Data source
SRFA	SRFA	NA	10 mg/L	DI, 4 M,1W, 256 scan, IAT 30/40 ms**.	Standard, this study.
SRFA_CBZ_2H	BLK_PHO	Photo-irradiation	MQW	LC, 2M, 1 W, 40 scans per minute, IAT 120 ms.	This study, experiment details in REF.[1,2]
	SRFA_PHO	Photo-irradiation	25 mg/L		
	SRFA_CBZ_PHO	Photo-irradiation	25 mg/L SRFA + CBZ 50 µM		
	SRFA_CBZ-D10_PHO	Photo-irradiation	25 mg/L SRFA + CBZ-D_{10} 50 µM		
EfOM_Oz_18O	Eff_18O	Ozonation	O_3/C = 0.5-1	LC, 2M, 1 W, 40 scans per minute, IAT 400 ms, AMP****.	REF.[2,3]
DW_Cl2	Drink_Cl2	Disinfection	4 mg/L Cl_2	LC, 2M, 1 W, 40 scans per minute, IAT 800 ms, CASI mode (m/z 150-250 Da).	REF.[4]
SRFA_Na	SRFA_Na_Adduct	NA	10 mg/L SRFA + 5 mg/L Na^+	DI, 8M, 1 W, 256 scans, IAT 25 ms.	This study

*MQW: Milli-Q water, conductivity of 18.2 MΩ/cm.
**IAT: Ion accumulation time.
***CASI: Continuous accumulation of selected ions (CASI) mode (also known as Q-isolation).
****AMP: Absorption mode processing.

Additional References:

(1) Raeke, J.; Lechtenfeld, O. J.; Seiwert, B.; Meier, T.; Riemenschneider, C.; Reemtsma, T. Photochemically Induced Bound Residue Formation of Carbamazepine with Dissolved Organic Matter. *Environ. Sci. Technol.* **2017**, *51* (10), 5523–5530. https://doi.org/10.1021/acs.est.7b00823.

(2) Han, L.; Kaesler, J.; Peng, C.; Reemtsma, T.; Lechtenfeld, O. J. Online Counter Gradient LC-FT-ICR-MS Enables Detection of Highly Polar Natural Organic Matter Fractions. *Anal. Chem.* **2021**, *93* (3), 1740–1748. https://doi.org/10.1021/acs.analchem.0c04426.

(3) Jennings, E. K.; Sierra Olea, M.; Kaesler, J. M.; Hübner, U.; Reemtsma, T.; Lechtenfeld, O. J. Stable Isotope Labeling for Detection of Ozonation Byproducts in Effluent Organic Matter with FT-ICR-MS. *Water Res.* **2023**, *229*, 119477. https://doi.org/10.1016/j.watres.2022.119477.

(4) Han, L.; Lohse, M.; Nihemaiti, M.; Reemtsma, T.; Lechtenfeld, O. J. Direct Non-Target Analysis of Dissolved Organic Matter and Disinfection By-products in Drinking Water with Nano-LC-FT-ICR-MS. *Environ. Sci.: Water Res. Technol.* **2023**, *9* (6), 1729–1737. https://doi.org/10.1039/D3EW00097D.

Performance of internal calibrations and robustness of median/mean value of M_{err} distribution.

Internal calibration was performed for every spectrum and all segments in DataAnalysis software, with known CHO series, yielding a root-mean-squared mass error (RMSE) of less than 0.2 ppm. For LC-FT-ICR MS measurements, the whole spectrum would be segmented by minutes and each segment was treated as an individual spectrum and internally calibrated, while the calibration results of segments with highest total ion intensity will be exported as calibration performance. Meanwhile, for experimental replicates, calibration results with highest RMSE will be used below as comparison.

Calibration performances of the 5 datasets were examined to evaluate the robustness and reproducibility of the applied internal calibration. Except blanks, all the measurements/datasets were calibrated with abundant calibrants cross the mass range, and yielded overall RMSE less than 0.2 ppm, most of them even below 0.1 ppm (Table S2).

For *SRFA_CBZ_2H* dataset, which has the most abundant multiple assignments (MultiAs), relative mass errors (RMEs) of calibrants still aligned well with the pre-set tolerance threshold (+/- 0.200 ppm) after internal calibration, as shown in Figure S1A. The averages of median and mean values of RMEs in this dataset were 0.004 (+/- 0.012) ppm and 0.006 (+/- 0.005) ppm, respectively, indicating overall excellent calibration performance and neglectable systematic error.

Meanwhile, the robustness of mean/median as proxy of true- and false-assignments were also examined. The M_{err} distributions of all internal calibrations from *SRFA_CBZ_2H* dataset was also checked, as shown in Figure S1B. The M_{err} of all internal calibrants were as small as 0.100 mDa, and the median and mean values are within 0.010 mDa (0.001 ± 0.003 mDa and 0.006 ± 0.004 mDa, respectively), demonstrating that systematic errors have been largely eliminated. Since they are smaller than the smallest mass differences that we have observed by now, medians/means are robust enough as references for recognizing false-assignments in groups caused by replacement pairs. Given that medians have lower standard deviation

(STD) than mean values, mainly because of the lower leverage of outliers, the medians of M_{err} were used for evaluation and comparison in this study.

Table S2. *Performance of internal calibrations.*

Dataset	Sample ID	Mass range (Da)	No. of calibrants	RMSE	Mean ppm
SRFA	SRFA	147-1000	323	0.100	0.008
SRFA_CBZ_2H	BLK_Pho	147-1000	13	0.063	0.001
	SRFA_PHO	147-1000	237	0.095	0.008
	SRFA_CBZ_PHO	147-1000	183	0.077	0.004
	SRFA_CBZ-D10_PHO	147-1000	206	0.100	0.008
EfOM_Oz_18O	Eff_18O	147-1000	> 140	<0.200	-
DW_Cl2	Drink_Cl2	150-250	> 58	<0.200	-
SRFA_Na	SRFA_Na_Adduct	147-1000	118	0.099	0.000

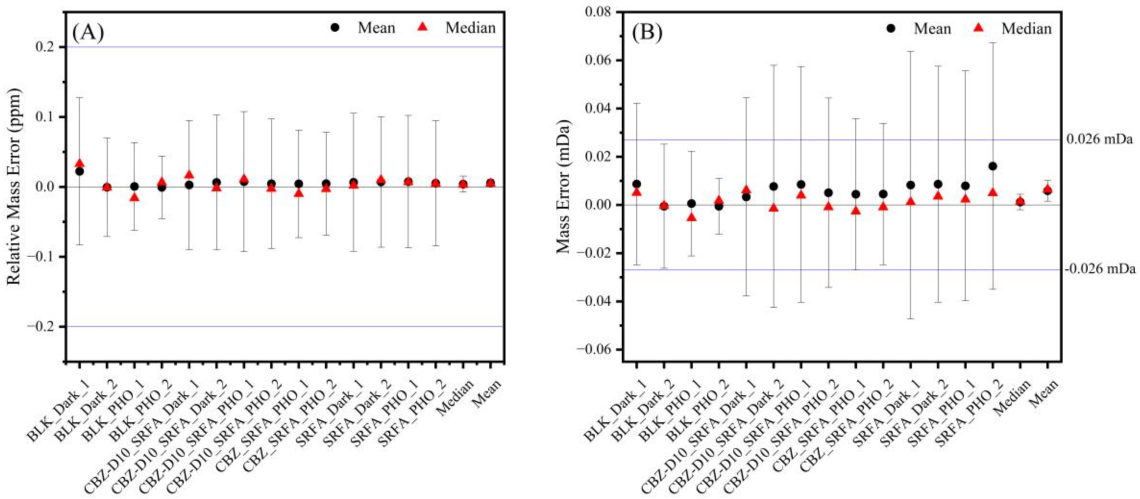

Figure S3. *Performances of internal calibrations from SRFA_CBZ_2H dataset: A) relative mass error in ppm; B) mass error in mDa. 0.026 mDa refers to the smallest mass difference of replacement pairs observed in SRFA datasets.*

Molecular formula assignments

Molecular formulas (MFs) were decided by chemical formula configuration (CFC), which includes elemental composition range and some experimental threshold. Except different elemental ranges described below, same thresholds of O/C ratio (0~1.2), H/C ratio (0.3~3), N/C ratio (0~1.5), DBE (0~25), and DBE-O (-10~10) were also applied for all dataset.

Table S3. Chemical formula configuration used for different dataset.

Dataset	CFC ID	Mass range (Da)	Element ranges	ppm tolerance
SRFA	CFC-N3S1	0-1000	"^{12}C": [1, 80],<br>"^{13}C": [0, 1],<br>"^{1}H": [0, 198],<br>"^{16}O": [0, 40],<br>"^{14}N": [0, 3],<br>"^{32}S": [0, 1],<br>"^{34}S": [0, 1]	± 0.5
	CFC-N5S3	0-1000	"^{12}C": [1, 80],<br>"^{13}C": [0, 1],<br>"^{1}H": [0, 198],<br>"^{16}O": [0, 40],<br>"^{14}N": [0, 5],<br>"^{32}S": [0, 3],<br>"^{34}S": [0, 1]	± 0.5
SRFA_CBZ_2H	CFC-N3S1D5	0-1000	"^{12}C": [1, 80],<br>"^{13}C": [0, 1],<br>"^{1}H": [0, 198],<br>"^{16}O": [0, 40],<br>"^{14}N": [0, 3],<br>"^{32}S": [0, 1],<br>"^{2}H": [0, 5],<br>"^{34}S": [0, 1]	± 1.0
EfOM_Oz_18O	CFC-N4S2-18O	0-1000	"^{12}C": [1, 80],<br>"^{13}C": [0, 1],<br>"^{1}H": [0, 198],<br>"^{16}O": [0, 40],<br>"^{14}N": [0, 4],<br>"^{32}S": [0, 2],<br>"^{18}O": [0, 5],<br>"^{34}S": [0, 1]	± 1.0
DW_Cl2	CFC-N2S1Cl3	0-1000	"^{12}C": [1, 80],<br>"^{13}C": [0, 1],<br>"^{1}H": [0, 198],<br>"^{16}O": [0, 40],<br>"^{14}N": [0, 2],<br>"^{32}S": [0, 1],<br>"^{35}Cl": [0, 3],	± 1.0

Dataset	CFC ID	Mass range (Da)	Element ranges	ppm tolerance
			"^{37}Cl": [0, 3],<br>"^{34}S": [0, 1]	
SRFA_Na	CFC-N5S3-Na	0-1000	"^{12}C": [1, 80],<br>"^{13}C": [0, 1],<br>"^{1}H": [0, 198],<br>"^{16}O": [0, 40],<br>"^{14}N": [0, 5],<br>"^{32}S": [0, 3],<br>"^{23}Na": [0, 1],<br>"^{34}S": [0, 1]	± 0.5

SRFA dataset

The *SRFA* dataset consists of duplicate measurements (i.e. SRFA-1 and SRFA-2) of Suwannee River Fulvic Acid (SRFA III, 3S101H) obtained with DI. The SRFA dataset underwent M_{err} distribution inspection as a whole.

Table S4. *Total number of formula assignments in SRFA dataset before automatic filtration.*

Chemical formula configuration	Sample ID	Total Peaks with Assignments	Total Number of formulas	Peaks with Multiple Assignments	Percent Multiple Assignments
CFC-N5S3	SRFA-1	14745	32157	9601	65
	SRFA-2	9989	19319	5547	56
	In total	24734	51476	15148	61 (average)
CFC-N3S1	SRFA-1	10469	11750	1243	12
	SRFA-2	7011	7833	817	12
	In total	17480	19583	2060	12 (average)

Table S5. *Main replacement pairs that cause multiple assignments (MultiAs) in SRFA dataset.*

Replacement pairs	Mass difference (mDa)	Related MultiAs with CFC-N5S3 (%)	Related MultiAs with CFC-N3S1 (%)
$H_8N_2S_3$ / C_7O_3	0.217	15.10	-
$H_3N_5S_2$ / $^{12}C_5{}^{13}C_1O_4$	0.026	10.27	-
$^{13}C_1H_1N_3O_4$ / C_{10}	0.060	10.12	84.16
$^{13}C_1H_5OS$ / C_2N_3	0.244	8.57	2.98
$C_7{}^{13}C_1{}^{34}S_1$ / $H_5N_1O_3S_2$	0.137	7.94	-
$^{13}C_1H_3N_1S_1{}^{34}S_1$ / O_6	0.354	5.98	-
$C_3H_7S_3$ / $^{13}C_1NO_7$	0.158	5.75	-
$C_2N_4{}^{34}S_1$ / H_2O_7	0.111	4.77	-
$C_2H_6S_2{}^{34}S_1$ / O_8	0.358	4.11	-
$^{13}C_1H_7N_3S_2{}^{34}S_1$ / C_8O_4	0.298	3.02	-
$C_9N_2{}^{34}S_1$ / $H_{10}O_4S_3$	0.107	2.49	-
$^{13}C_1N_1{}^{34}S_1$ / $C_1H_1O_1S_1$	0.515	2.61	2.95
$C_{10}H_2S_1{}^{34}S_1$ / N_2O_{10}	0.294	1.93	-
$C_9{}^{34}S_1$ / $H_2N_2O_5S_1$	0.575	1.53	1.07
$H_4N_2O_2S$ / C_8	0.651	1.31	3.26
$H_6O_6{}^{34}S_1$ / $C_4N_4S_1$	0.062	1.85	-
N_4O_1 / H_8S_2	0.468	1.22	-
$H_9N_1O_2S_1{}^{34}S_1$ / $C_9{}^{13}C_1$	0.088	1.12	-
SUM		89.69	94.42

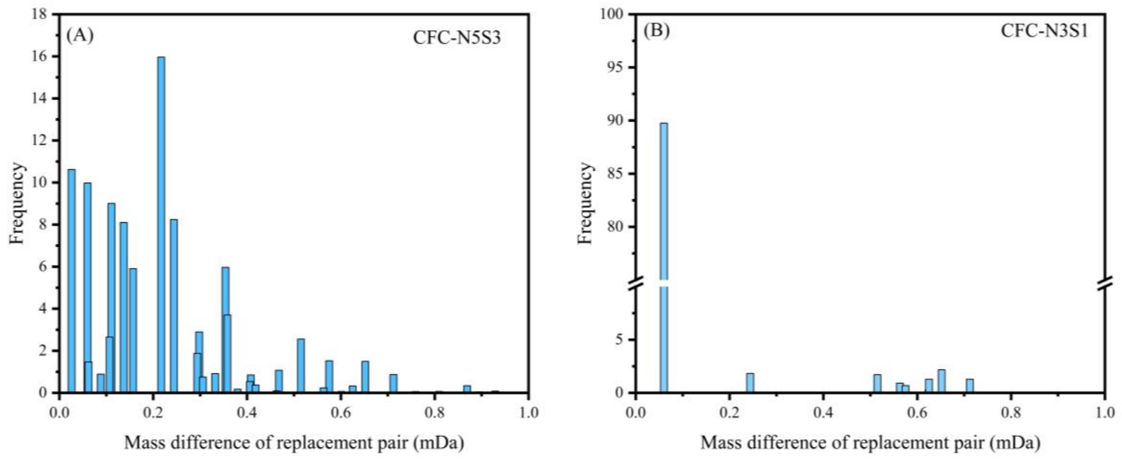

Figure S4. *Frequency of replacement pairs in MultiAs observed in SRFA dataset with 2 different CFC: (A) MultiAs caused by replacement pairs from CFC-N5S3; (B) MultiAs caused by replacement pairs from CFC-N3S1.*

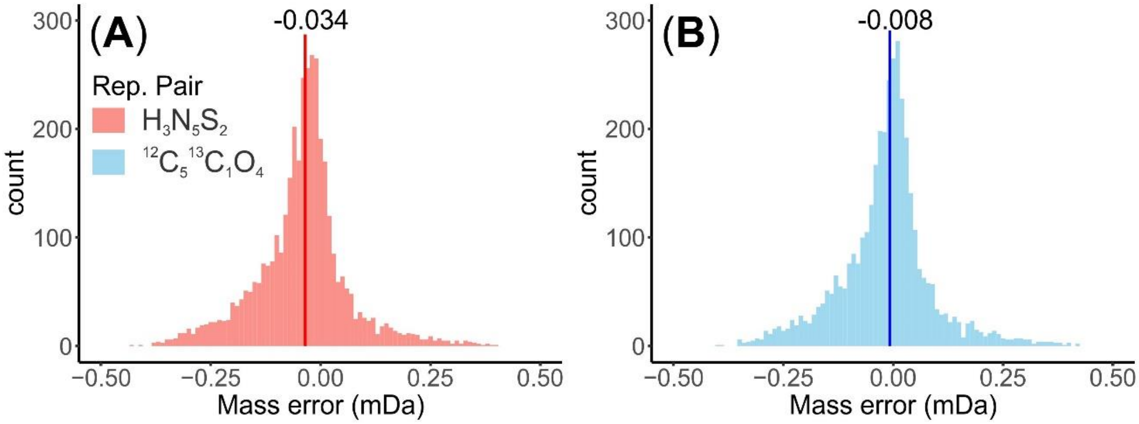

Figure S5. *Example multiple assignment and its replacement pair in SRFA dataset ($H_3N_5S_2$ vs $^{12}C_5{}^{13}C_1O_4$, 0.026 mDa mass difference, n =7640): (A) M_{err} distribution of false-assignments "N5S2" molecular formula (MF) (with median of -0.034 mDa) from Figure 2(A); (B) M_{err} distribution of true-assignments "^{13}CHO" MF (with median value of -0.008 mDa) from Figure 2(A).*

Table S6. *Total number of formula assignments in SRFA dataset after automatic filtration.*

Chemical formula configuration	Sample ID	Total Number of formulas	Peaks with Assignments	Peaks with Multiple Assignments	Percent Multiple Assignments
CFC-N5S3	SRFA-1	14938	14745	193	1.3
	SRFA-2	10171	9989	182	1.7
	In total	25109	24734	375	1.5
CFC-N3S1	SRFA-1	10473	10469	4	< 0.1
	SRFA-2	7018	7011	7	0.1
	In total	17491	17480	11	< 0.1

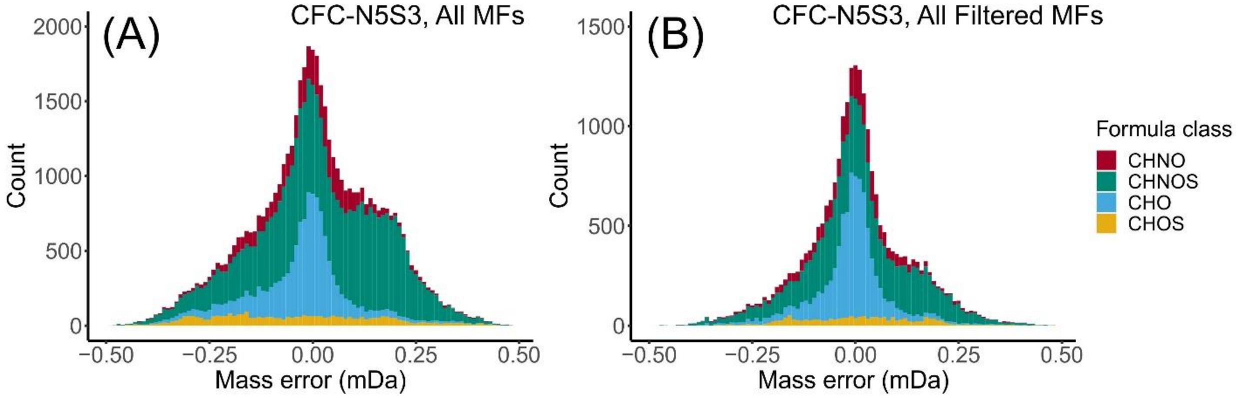

Figure S6. *M_{err} distribution of SRFA dataset: (A) all formulas before filtration; (B) formulas with MultiAs filtered by M_{err} inspection subset from KMD-CH_2 and formula classes.*

SRFA_Na Dataset

When DOM samples are not desalted enough, some Na adducts might occur even though samples are measured in negative mode, which were affirmed by fragmentation spectrum (Figure S6). Duplicate SRFA samples with 5 mg/L Na^+ were measured in negative mode, after which ions were neutralized by adding a proton and were assigned formulas with CFC-N5S3-Na.

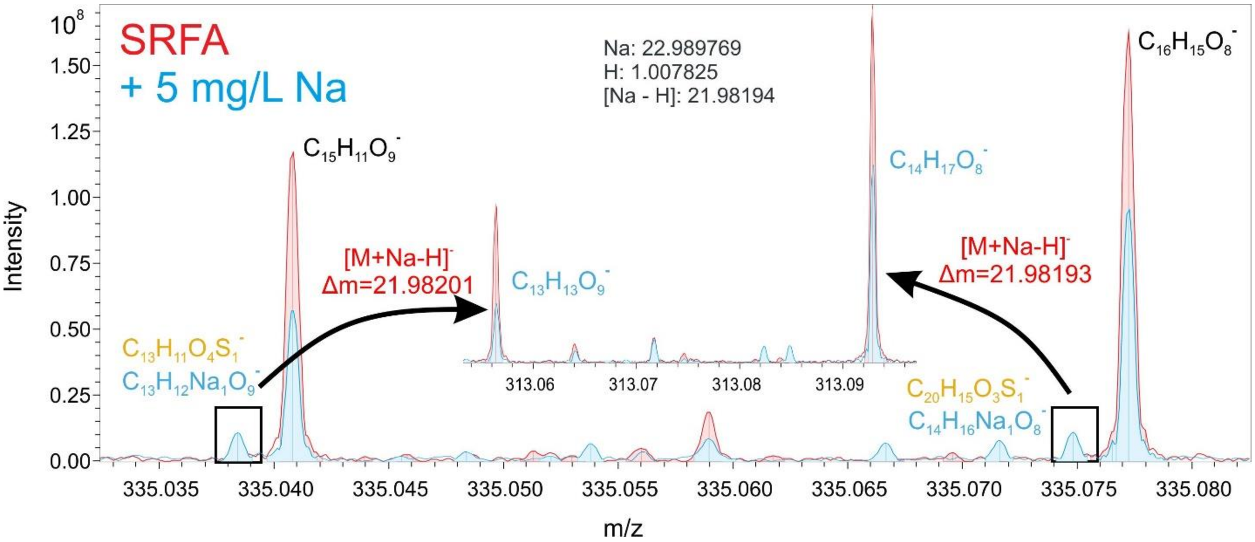

Figure S7. *Expanded section from a full scan mass spectrum showing Na^+ adducts in SRFA_Na (blue; m/z 335.03846: $[C_{13}H_{12}Na_1O_9]^-$ and m/z 335.07487: $[C_{14}H_{16}Na_1O_8]^-$) measured with ESI negative mode. These peaks are not present in SRFA (i.e., without NaCl added, red). Inset shows mass peaks at m/z 313.05645: $[C_{13}H_{13}O_9]^-$ and m/z 313.09294: $[C_{14}H_{17}O_8]^-$ corresponding to the deprotonated form of the Na^+-adducts. The peak magnitude of the deprotonated species decreases upon addition of Na. The Na^+-adducts also have a multiple assignment in form of a highly unsaturated and oxygen-poor S-containing molecular formula ($[C_{19}H_{11}O_4S_1]^-$ and $[C_{20}H_{15}O_3S_1]^-$), which may be the only assignment if Na is not considered.*

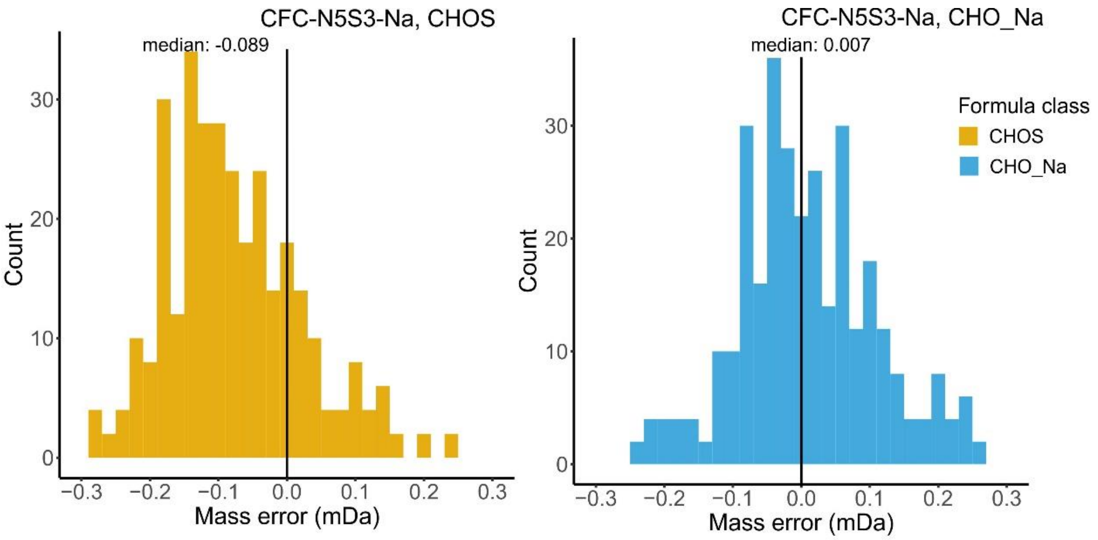

Figure S8. *Mass error distribution of MultiAs caused by Na^+ adducts in SRFA_Na dataset (C_6S vs HO_5Na, 0.096 mDa difference in mass): (A) M_{err} distribution of false-positive C_6S molecular formula (MF) (with median of -0.089 mDa); (B) M_{err} distribution of true-positive HO_5Na MF (with median value of 0.007 mDa).*

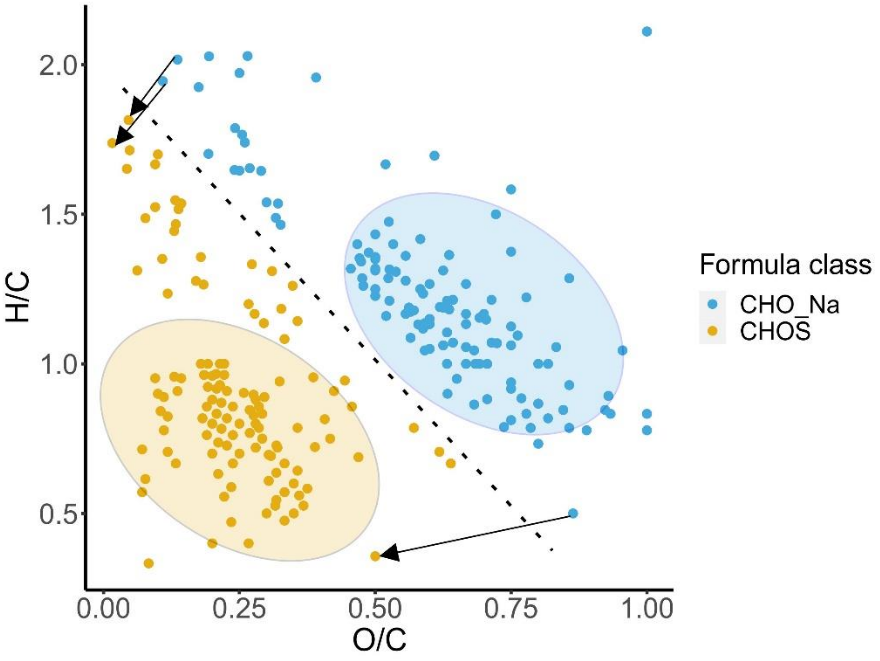

Figure S9. *van Krevelen plot of MultiAs caused by replacement pair of C_6S (CHOS molecular formula (MF)) vs HO_5Na (CHO_Na MF) in SRFA_Na dataset. Arrows indicate changes in O/C and H/C between false-assigned CHOS formulas (sometimes referred to as "black sulfur") and true-assigned CHO sodium adducts.*

Multiple assignments caused by O_1P_1 / $C_1{}^{35}Cl_1$

Tolić et.al reported multiple assignments caused by replacement pair O_1P_1 / $C_1{}^{35}Cl_1$ with mass difference of 0.176 mDa.[1] The CHOCl MFs were validated by better agreement of ppm error distribution with calibrants.[1] When P was considered, 388 peaks in *DW_Cl2* dataset had MultiAs caused by this replacement pair mass, and the error distribution of MultiAs was re-assessed in mDa.

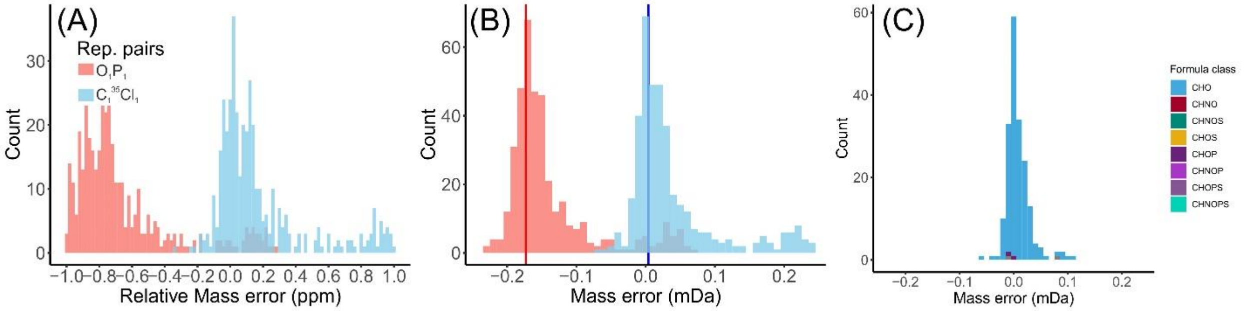

Figure S10. *Mass error distribution of molecular formulas (MFs) related to O_1P_1 / $C_1{}^{35}Cl_1$ ("CHOP" MF vs. "CHOCl" MF, 0.176 mDa difference in mass) in the DW_Cl2 dataset: (A) overlapped mass error distribution of multiple assignments in ppm; (B) overlapped mass error distribution of multiple assignments in mDa and (C) data filtered by M_{err} inspection in homologous groups (here CHO referes to CHOCl MF class). Note that 2 P-containing MF were retained after filtration.*

Additional Reference:

(1) Tolić, N.; Liu, Y.; Liyu, A.; Shen, Y.; Tfaily, M. M.; Kujawinski, E. B.; Longnecker, K.; Kuo, L.-J.; Robinson, E. W.; Paša-Tolić, L.; Hess, N. J. Formularity: Software for Automated Formula Assignment of Natural and Other Organic Matter from Ultrahigh-Resolution Mass Spectra. *Anal. Chem.* **2017**, *89* (23), 12659–12665.

EfOM_Oz_18O dataset

Wastewater treatment plant effluent (EfOM) samples were oxidized with heavy ozone ($^{18}O_3$, 50% purity), after which organic matter was isolated by solid phase extraction and measured by DI-FT-ICR-MS. Spectra were processed using absorption mode processing to improve mass accuracy after acquisition. EfOM_Oz_18O dataset consists of 2 samples including ozonated (EfOM_Oz) and unozonated EfOM and was used here for analysis of MultiAs. Molecular formulas were regulated with harsh RME threshold of ± 0.2 ppm, after which the ^{34}S and ^{13}C isotopologue peak abundance were validated.

Table S7. *Total number of formula assignments in EfOM_Oz_18O dataset before automatic filtration.*

Chemical formula configuration	Sample ID	Total Peaks with Assignments	Total Number of formulas	Peaks with Multiple Assignments	Percent Multiple Assignments
CFC-N5S3-18O	EfOM_Oz	5096	5368	271	5
	EfOM_nOz	8804	9438	634	7
	In total	13900	14806	905	6 (average)

Table S8. *Main replacement pairs that cause multiple assignments (MultiAs) in EfOM_Oz_18O.*

Replacement pairs	Mass difference (mDa)	Related MultiAs	Related MultiAs (%)
$^{18}ON_2$ / CH_2O_2	0.172	1696	93.6
$C_7H_2{}^{18}O_1S_1$ / N_4O_5	0.013	72	4.0
$C_6{}^{18}O_2S_1$ / N_2O_7	0.157	16	0.9
SUM		1784	98.5

Table S9. *Total number of formula assignments in EfOM_Oz_18O dataset after workflow filtration. MF = Molecular formula.*

Chemical formula configuration	Sample/dataset ID	Total Number of formulas	Verified Assignments	Peaks with Multiple Assignments	Fraction of Peaks with MultiAs to all Assigned Peaks
CFC-N5S3-18O	EfOM_Oz	5096	5096	NA	-
	EfOM_nOz	8804	8804	NA	-
	In total	13900	13900	NA	-
CFC-N5S3-18O	$^{18}ON_2$ MFs	848	582		
	CH_2O_2 MFs	848	266		

Performance of workflow for data filtering

In this study, specificity, accuracy and precision were assessed on and MultiAs caused only by replacement pair ($H_3N_5S_2$ / $^{12}C_5{}^{13}C_1O_4$) in *SRFA* dataset which has been systematically investigated. Those MFs with $^{12}C_5{}^{13}C_1O_4$ were condition positives (P), while MFs with $H_3N_5S_2$ were condition negatives (N). The intersection between T/P and test outcome positives/negatives were used, i.e. true positives (TP), true negative (TN), false positive (FP) and false negative (FN).

Accuracy (ACC) = $\frac{(TP+TN)}{(P+N)}$

Precision, positive predictive value (PPV) = $\frac{TP}{(TP+FP)}$

Sensitivity, true positive rate (TPR) = $\frac{TP}{(TP+FN)}$

Specificity, true negative rate (TNR) = $\frac{TN}{(TN+FP)}$

The values were calculated and listed below (Table S10).

Table S10. *Performance of automatic data filtering algorithm for SRFA dataset. MF = Molecular formula.*

Replacement pairs	Retained in SRFA filtered data by workflow	Removed from SRFA data by workflow	SUM	
MFs with $^{12}C_5{}^{13}C_1O_4$ in SRFA dataset (P)	2244 (TP)	1576 (FN)	3820	TPR (59%)
MFs with $H_3N_5S_2$ in SRFA dataset (N)	575 (FP)	3245 (TN)	3820	TNR (85%)
SUM	2819	4821	7640 (P+N)	
	PPV (80%)		ACC (72%)	

Table S11. *Gaussian distribution fitting of M_{err} in SRFA dataset.*

Annotations	Median (mDa)	Mean (m Da)	SD (σ)
All filtered formula	-0.001	0.002	0.124
Formulas with $^{12}C_5{}^{13}C_1O_4$	-0.008	-0.016	0.105
Formulas with $H_3N_5S_2$	-0.034	-0.042	0.105
Mass difference of $H_3N_5S_2$ and $^{12}C_5{}^{13}C_1O_4$	0.026	0.026	-
Minimum datapoint for estimation of mean/median of 0.026 mDa*		130	

*Datapoints are estimated by Lehr's equation as follow:

$$n = \frac{8}{(\Delta)^2}$$

where standardized difference is $\Delta = (\mu_0 - \mu_1)/\sigma$. And the distribution is compared with known population value ($\mu_0 = 0$) and n is calculated in the one-sample case ($\alpha = 0.05$).

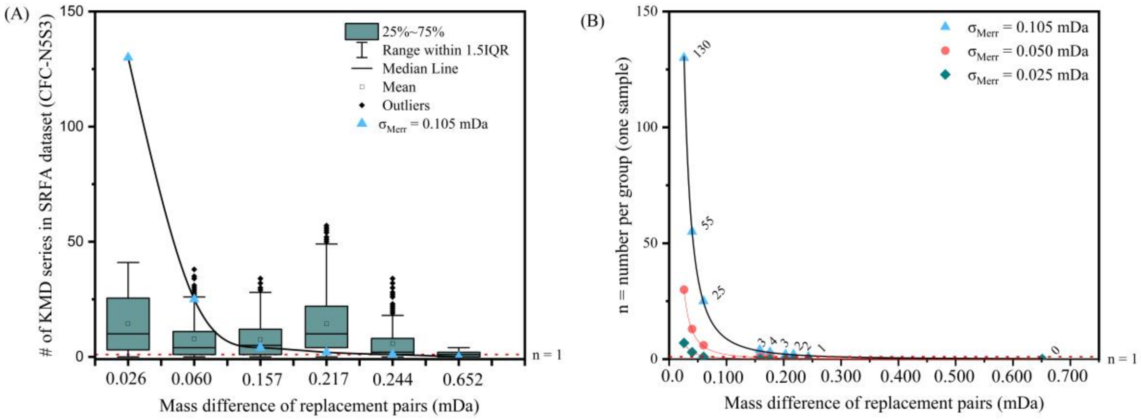

Figure S11. *Sample size needed for proper estimation of M_{err} distribution of different replacement pairs and number of KMD series in the SRFA dataset (A) and (B) sample size estimation with different SD (according to instrumental mass accuracy).*

DW_Cl2 dataset

Table S12. *Total number of formula assignments in DW_Cl2 dataset before automatic filtration.*

Chemical formula configuration	Sample ID	Total Peaks with Assignments	Total Number of formulas	Peaks with Multiple Assignments	Percent Multiple Assignments
CFC-N2S1Cl3	Cl2_4mg_FD_CASI _150_250_13_14m	1876	3137	732	39
	Cl2_4mg_FD_CASI _150_250_14_15m	1904	3130	728	38
	In total	3780	6267	1460	38.6 (ave.)

Table S13. *Dominant replacement pairs that cause multiple assignments (MultiAs) in DW_Cl2 dataset.*

Replacement pairs	Mass difference (mDa)	Related MultiAs (%)
$CH_2{}^{37}ClS$ / ClO_3	0.027	31.74
$H_3O_4S_1$ / C_3ClN_2	0.203	5.74
$H_6{}^{37}Cl_2N_2$ / C_5O_3	0.160	4.70
HO_7 / $C_4{}^{37}ClN_2$	0.176	4.48
$H_9Cl_2{}^{37}Cl_1$ / C_3O_5	0.540	3.24
NO_3 / ${}^{12}C^{13}CH_2Cl$	0.040	2.41
${}^{37}Cl_1O_2$ / $H_2Cl_1S_1$	0.842	2.41
$H_4Cl_1{}^{37}Cl_1N_2$ / C_6S_1	0.132	2.04
$H_7{}^{37}Cl_1O_4$ / C_9	0.336	2.02
Cl_2O_1 / $C_1{}^{37}Cl_2$	0.815	1.82
$C_3H_2{}^{37}Cl_2$ / O_5S_1	0.811	1.48
${}^{37}Cl_1N_2O_2$ / $C_2H_3Cl_2$	0.700	1.48
${}^{13}C_1Cl_2$ / ${}^{37}C_1N_1S_1$	0.012	1.15
$H_9Cl_1{}^{37}Cl_2S_1$ / C_2O_8	0.513	1.43
SUM		66.14

Table S14. *Total number of formula assignments in DW_Cl2 dataset after automatic filtration.*

Chemical formula configuration	Sample ID	Total Number of formulas	Peak with Assignments	Peaks with Multiple Assignments	Fraction of Peaks with MultiAs to all Assigned Peaks
CFC-N2S1Cl3	Cl2_4mg_FD_CASI 150_250_13_14m	1876	1876	NA	-
	Cl2_4mg_FD_CASI 150_250_14_15m	1904	1904	NA	-
	In total	3780	3780	NA	-

Table S15. *Total number of Chlorine formula assignments in DW_Cl2 dataset before and after filtration.*

	Before filtration			After filtration		
Dataset	Total Cl formulas	Total ^{35}Cl monoisotopologues	Total $^{35}Cl/^{37}Cl$ pairs	Total Cl formulas	Total ^{35}Cl monoisotopologues	Total $^{35}Cl/^{37}Cl$ pairs
DW_Cl2	4334	1466	539	1991	969	395

Table S16. *Performance of automatic data filtering algorithm for DW_Cl2 dataset. MF = Molecular formula.*

Replacement pairs	Retained in DW_Cl2 filtered data by workflow	Removed from DW_Cl2 data by workflow	SUM	
Cl-MFs in MultiAs validated by isotope filter (P)	98(TP)	26 (FN)	124	TPR (79%)
Related false-assignment rejected by isotope filter (N)	27 (FP)	111 (TN)	138	TNR (80%)
SUM	125	137	262 (P+N)	
	PPV (78%)	-	ACC (80%)	

Table S17. *MultiAs caused by $CH_2{}^{37}ClS$ and ClO3 in DW_Cl2 dataset during Merr validation. MF = Molecular formula.*

	Before filtration			After filtration				
Dataset	Total Multi As	ClO_3 MFs have ^{37}Cl isotopologues	$CH_2{}^{37}ClS$ have ^{35}Cl monoisotopologues	Total MFs retained	ClO_3 MFs	$CH_2{}^{37}ClS$ MFs	ClO_3 MFs have ^{37}Cl isotopologues	$CH_2{}^{37}ClS$ have ^{35}Cl monoisotopologues
MultiAs from $CH_2{}^{37}ClS$ and ClO_3	950	361	9	463	362	101	270	4

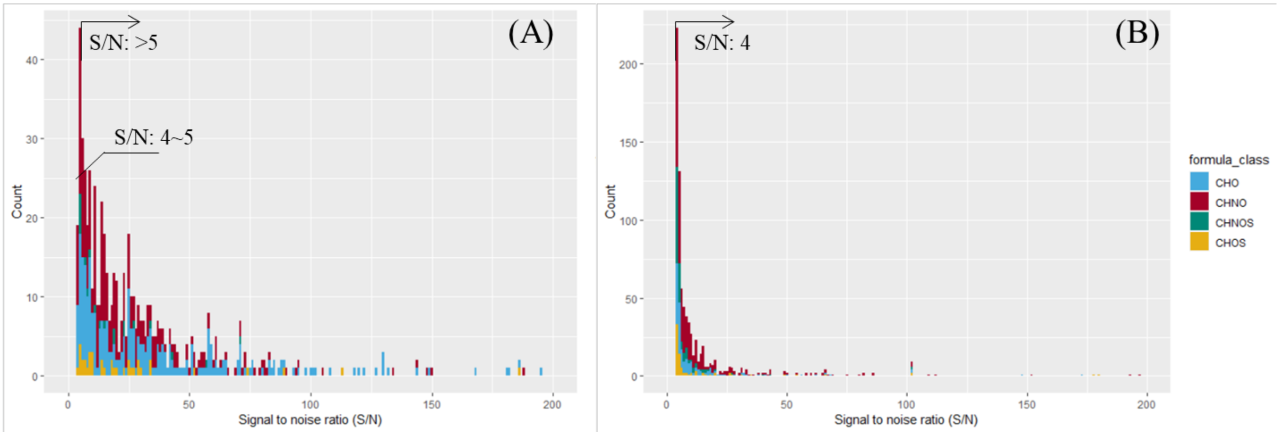

Figure S12. *S/N distributions of ^{35}Cl formulas before data filtering (plotted with bin size of 1): (A) ^{35}Cl formulas matched with ^{37}Cl isotopologues; (B) ^{35}Cl formulas matched with no ^{37}Cl isotopologues.*

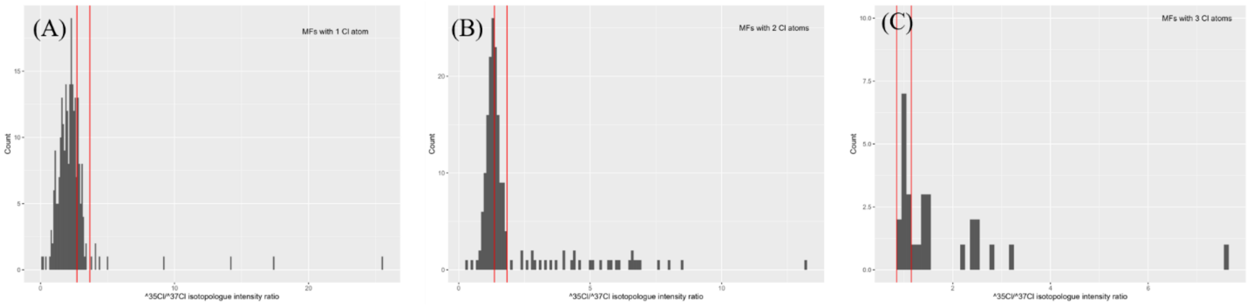

Figure S13. *Initial $^{35}Cl/^{37}Cl$ isotopologue intensity ratio: (A) molecular formulas (MFs) with one Cl atom (($^{35}Cl_1{}^{37}Cl_0$)/ ($^{35}Cl_0{}^{37}Cl_1$) = 3.2 ± 15%, red lines at 15% tolerance); (B) MFs with two Cl atoms (($^{35}Cl_2{}^{37}Cl_0$)/ ($^{35}Cl_1{}^{37}Cl_1$) = 1.6 ± 15%, red lines at 15% tolerance); (C) MFs with three Cl atoms (($^{35}Cl_3{}^{37}Cl_0$)/ ($^{35}Cl_2{}^{37}Cl_1$) = 1.0 ± 15%, red lines at 15% tolerance).*

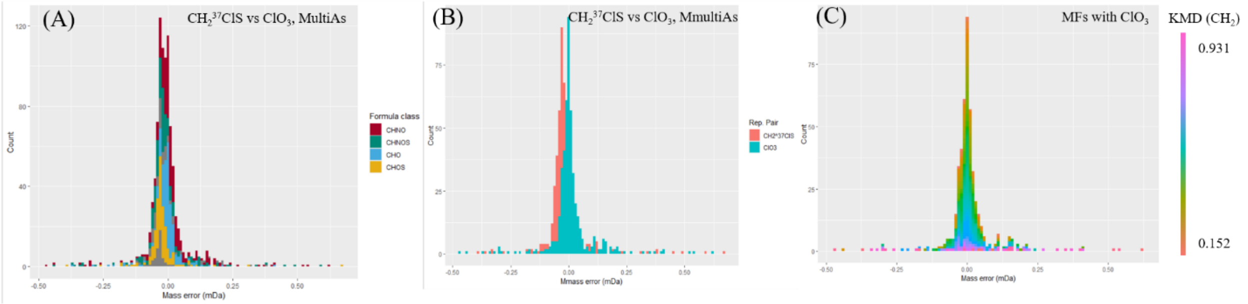

Figure S14. *Mass error distribution of ^{37}Cl dataset ($CH_2{}^{37}ClS$ and ClO_3, 0.027 mDa): (A) & (B) multiple assignments from this replacement pair; (B) M_{err} distribution with KMD-CH_2 of ClO_3 molecular formulas (MFs).*

Additional Reference:

(1) Han, L.; Lohse, M.; Nihemaiti, M.; Reemtsma, T.; Lechtenfeld, O. J. Direct Non-Target Analysis of Dissolved Organic Matter and Disinfection By-products in Drinking Water with Nano-LC-FT-ICR-MS. *Environ. Sci.: Water Res. Technol.* **2023**, *9* (6), 1729–1737. https://doi.org/10.1039/D3EW00097D.

SRFA_CBZ_2H dataset

Table S18. *Dominant replacement pairs that cause multiple assignments (MultiAs) in one measurement in SRFA_CBZ_2H dataset (measurement: CBZ_D10_SRFA_pho_1_17_18min).*

Replacement pairs	Mass difference (mDa)	Related MultiAs (%)
D_2S_1 / C_3	0.275	18.90
$C_2D_1O_1$ / N_3	0.206	14.31
$D_1N_3S_1$ / C_5O_1	0.480	7.23
D_2O_5 / $C_4H_4S_1$	0.594	5.95
D_4O_5 / C_7H_4	0.320	5.59
$^{13}C_1H_5$ / $^{12}C_1D_3$	0.175	4.52
$D_3O_1S_1$ / C_1N_3	0.069	4.51
D_2N_3 / $^{12}C_1{}^{13}C_1H_5O_1$	0.031	4.42
$D_3N_3O_4$ / C_9H_4	0.114	4.13
D_5S_1 / $^{12}C_2{}^{13}C_1H_5$	0.100	4.00
$D_1N_3O_4$ / $C_6H_4S_1$	0.389	3.67
$D_3N_1O_2$ / $C_1H_8S_1$	0.537	3.16
$C_1D_4O_3$ / $H_8N_2S_1$	0.332	2.51
$D_4N_3S_1$ / $^{12}C_4{}^{13}C_1H_5O_1$	0.305	2.46
$D_5N_1O_2$ / C_4H_8	0.812	2.05
C_5D_2 / $H_4N_2O_2$	0.926	1.54
D_3O_6 / $C_2H_4N_3S_1$	0.800	1.32
SUM		90.27

Table S19. *Total number of formula assignments in SRFA_CBZ_2H dataset before and after filtration. MF = Molecular formula.*

Chemical formula configuration	Total Peaks with Assignments	Total Number of formulas	Peaks with Multiple Assignments	Percent Multiple Assignments	MFs with deuterium atoms	Comments
CFC-N3S1D5	163265	447416	113535	69.5	302262	Before filtration
CFC-N3S1D5	131214	131214	NA	-	43124	After filtration

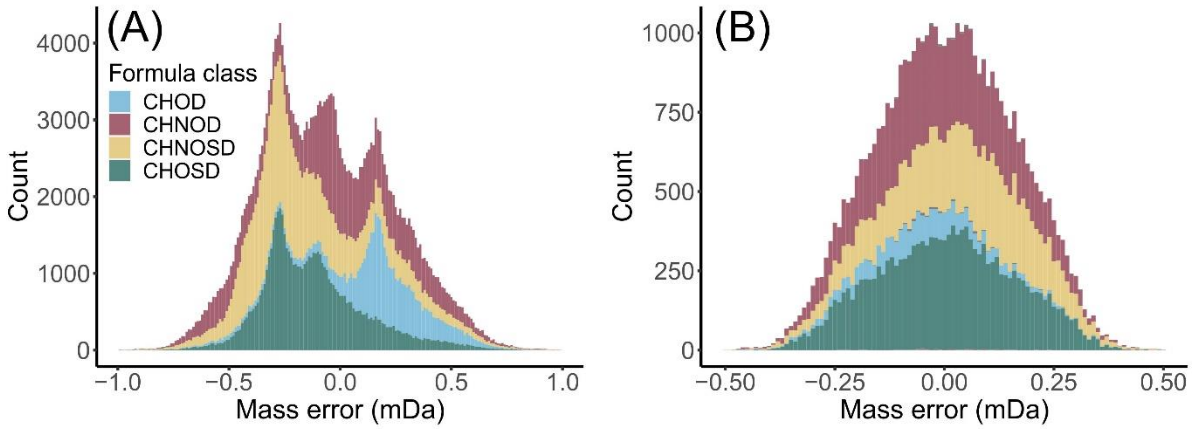

Figure S15. *Mass error distribution of ^2H formulas: (A) ^2H formulas in multiple assignments; (B) Multiple assignments filtered by M_{err} inspection.*

***Table S20**. Duration reported when running R script snippet for different data inputs. Results were tested on laptop with CPU of Intel-i7, and SSD of 512 GB, R version 4.2.1.*

Data file	Observation of all formula	Observation of multiple assignments	Duration reported (**Seconds**)
SRFA_formulas_CFC_N3S1.csv	19,583	4,263	9.51
SRFA_Na_adducts.csv	87,659	77,473	10.15
EfOM_Oz_18O.csv	14,806	1,857	10.24
DW_Cl2.csv	6,267	4,014	11.77
CHOCl_CHOP.csv	21,211	20,075	13.68
SRFA_formulas_CFC_N5S3.csv	91,373	85,694	14.10
SRFA_CBZ_2H.csv	435,308	394,791	27.80
